# Identification of the mechanisms that drive the toxicity of TiO_2 _particulates: the contribution of physicochemical characteristics

**DOI:** 10.1186/1743-8977-6-33

**Published:** 2009-12-17

**Authors:** Helinor J Johnston, Gary R Hutchison, Frans M Christensen, Sheona Peters, Steve Hankin, Vicki Stone

**Affiliations:** 1 Centre for Nano Safety, School of Life Sciences, Edinburgh Napier University, Edinburgh EH10 5DT, UK; 2 Department for Environment, Food and Rural Affairs, Chemicals and Nanotechnologies Division, Area 2a Nobel House, 17 Smith Square, London SW1P 3JR, UK; 3 Nanobiosciences unit, European Commission - DG Joint Research Centre (JRC), Institute for Health and Consumer Protection (IHCP), Via E. Fermi, 2749, I - 21027 Ispra, Italy; 4 Institute of Occupational Medicine, Research Avenue North, Riccarton, Edinburgh, EH14 4AP, UK

## Abstract

This review focuses on outlining the toxicity of titanium dioxide (TiO_2_) particulates *in vitro *and *in vivo*, in order to understand their ability to detrimentally impact on human health. Evaluating the hazards associated with TiO_2 _particles is vital as it enables risk assessments to be conducted, by combining this information with knowledge on the likely exposure levels of humans. This review has concentrated on the toxicity of TiO_2_, due to the fact that the greatest number of studies by far have evaluated the toxicity of TiO_2_, in comparison to other metal oxide particulates. This derives from historical reasons (whereby the size dependency of particulate toxicity was first realised for TiO_2_) and due to its widespread application within consumer products (such as sunscreens). The pulmonary and dermal hazards of TiO_2 _have been a particular focus of the available studies, due to the past use of TiO_2 _as a (negative) control when assessing the pulmonary toxicity of particulates, and due to its incorporation within consumer products such as sunscreens. Mechanistic processes that are critical to TiO_2 _particulate toxicity will also be discussed and it is apparent that, in the main, the oxidant driven inflammatory, genotoxic and cytotoxic consequences associated with TiO_2 _exposure, are inherently linked, and are evident both *in vivo *and *in vitro*. The attributes of TiO_2 _that have been identified as being most likely to drive the observed toxicity include particle size (and therefore surface area), crystallinity (and photocatalytic activity), surface chemistry, and particle aggregation/agglomeration tendency. The experimental set up also influences toxicological outcomes, so that the species (or model) used, route of exposure, experiment duration, particle concentration and light conditions are all able to influence the findings of investigations. In addition, the applicability of the observed findings for particular TiO_2 _forms, to TiO_2 _particulates in general, requires consideration. At this time it is inappropriate to consider the findings for one TiO_2 _form as being representative for TiO_2 _particulates as a whole, due to the vast number of available TiO_2 _particulate forms and large variety of potential tissue and cell targets that may be affected by exposure. Thus emphasising that the physicochemical characteristics are fundamental to their toxicity.

## Introduction

The field of nanotechnology is expanding at a tremendous rate due to the realisation that the properties exhibited by materials at a 'nano' scale are often exceedingly different to those demonstrated by bulk forms of the same material. Nanomaterials (defined as having at least one dimension less than 100 nm [1]), of various types and quantities, are therefore attractive for exploitation within diverse products, which harness the novel properties exhibited by materials with nano dimensions. As a result, an improved understanding of the potential risks (comprising of exposure and hazard assessments) of such materials is required, and in particular, determination of nanomaterial characteristics that may detrimentally affect human health (see for example, Maynard et al. [2]). This knowledge will be useful in managing risk in the future, by allowing the implementation of specific control measures for minimising exposure to such materials, perhaps through the introduction of regulations, or through the use of alternative materials. This would therefore allow safety to be built into the design of nanomaterials and their applications, to allow their safe integration into products. This hazard review, relating to the toxicity of titanium dioxide (TiO_2_) particulates was adapted from a series of reviews conducted as part of the Engineered Nanoparticles: Review of Health and Environmental Safety' (ENRHES) project, funded by the European Commission FP7 funding programme http://nmi.jrc.ec.europa.eu/project/ENRHES.htm. The project aimed to conduct a comprehensive, and critical review of the available health and environmental safety data for a variety of nanomaterials, in order to determine the current level of understanding (relating to nanomaterial toxicity to humans), and to identify the current gaps in knowledge, thereby allowing elucidation of the research that should be conducted with highest priority in the future. In addition, the hazard information generated within this review is to be combined with a review of the available human and environmental exposure data and an evaluation of industrial activity in this area, in order to provide the basis for a risk assessment, based on current knowledge.

Metal oxide nanoparticles (NPs) can be composed of a variety of diverse materials, including titanium, zinc, cerium, aluminium and iron oxides. The size of such particles is integral to their exploitation, but size is also responsible for prompting concern surrounding their potential toxicity. When considering metal oxide particulates, the greatest number of available studies have focussed on revealing the toxicity of TiO_2_, due to its widespread exploitation. These studies will therefore constitute the focus of this review. TiO_2 _exposure, via the pulmonary route, has been considered with particular interest due to its use as a (negative) control particulate when assessing the pathogenicity of other particulate materials such as alpha-quartz (see below), but also due to concern regarding occupational exposures. Assessing TiO_2 _particle toxicity to the skin has also formed the focus of a number of studies, due to their inclusion within sunscreens and cosmetic products. The ingestion of TiO_2 _is also of relevance due to its incorporation into foods and medicines.

Concern about the potential toxicity of nanoparticles in general, and TiO_2 _in particular, originally emanates from the studies conducted by Ferin et al. [3,4] and Oberdorster et al. [5]. These investigators first demonstrated that pulmonary inflammation, particle retention and translocation of ultrafine (equivalent to NPs, in terms of size) TiO_2 _was enhanced, compared to that of its larger, fine equivalent. Previously, TiO_2 _has been largely used in pulmonary toxicology studies as a negative control when assessing the toxicity of pathogenic particulates such as alpha-quartz. As a result, this had to be re-considered based on the apparent size dependency of TiO_2 _toxicity. These studies paved the way for the consideration of particle size, in relation to the toxic response that is manifested by particulates. More recently, studies have continued to focus on the importance of TiO_2 _particle size (and surface area) to particle toxicity, but have now expanded considerations to include determining the contribution of the particle crystal phase, surface chemistry, and photoactivity to their toxic potential.

## *In vivo *assessment of TiO_2 _toxicity

In general, *in vivo *investigations that evaluate the toxicity of TiO_2 _particles concentrate on a particular route of exposure, which is driven by the anticipated application of TiO_2_, for example the utilisation of TiO_2 _containing sunscreens has prompted investigations into the dermal toxicity of TiO_2_.

## Pulmonary exposure to TiO_2 _particulates

The greatest number of available studies, by far address the consequences of the exposure of the lungs to TiO_2_. In particular, the size dependence of any effects has been prioritised within available investigations. In addition, consideration of the contribution of the experimental set up to the observed toxicity has also been a focus of a number of studies. A summary of the pulmonary toxicity of TiO_2 _can be found in table 1.

**Table 1:** The pulmonary toxicity exhibited by TiO_2_

Paper	Particle	Model	Endpoints	Findings
Ahn et al., [14]	TiO_2 _(0.29 μm)	Intratracheal Instillation (4-72 hour exposure) -rats	BALF cell infiltration Number of goblet cells Muc5ac expression (indicative of mucus secretion) IL-13 production Histology (for lung tissue morphology)	Increased neutrophils, eosinophils & goblet cells Increased Muc5ac expression Increased IL-13

Renwick et al., [6]	TiO_2 _(29 & 250 nm)	Intratracheal Instillation (24 hour exposure) -rats	Inflammation (BALF analysis) Epithelial cell damage Lung permeability (BALF protein) Cytotoxicity (BALF LDH) Macrophage phagocytic ability (determining the uptake of fluorescent polystyrene particles) Macrophage chemotaxis (ability to migrate towards C5a)	NPs induce a neutrophil infiltration NPs damage epithelial cells Increased Lung permeability Increased Cytotoxicity NPs impair macrophage phagocytosis NP treated macrophages increased chemotaxis

Chen et al., [12]	TiO_2 _(18-21 & 180-250 nm)	Intratracheal Instillation (3 day to 2 week exposure) -mice	Morphological analysis (included investigation of enlarged alveoli, disrupted septa, thickened alveoli) Apoptosis in lung tissue (TUNEL assay) Immunohistochemical staining (antiPCNA) cDNA microarray analysis rtPCR & Western Blot (for placenta growth factor (P1GF))	Morphology of lung injury was emphysema-like for NPs. Observed macrophage infiltration that were particle laden Increased apoptosis in lung tissue Gene expression (chemokines & complement) changes indicative of an inflammatory response P1GF (a cytokine inducer) expression anticipated to be central to the inflammatory response • No pathology observed for fine particles

Warheit et al., [11]	TiO_2 _(in various crystal forms)	Intratracheal instillation (24 hours to 3 month exposure) -rats	Inflammation (BALF cells & cytokines) Lung permeability (BALF protein) Cytotoxicity (BALF LDH) Epithelial cell secretory activity (alkaline phosphatase) Lung histopathology	Neutrophil infiltration No cytotoxicity, protein, alkaline phosphatase and lung morphology changes Macrophage accumulation but normal • Crystallinity of sample impacts on pulmonary toxicity (greater toxicity for anatase containing particles)

Bermudez et al., [8]	TiO_2 _(1.40 μm)	Inhalation (13 week exposure) -mice, rats, hamsters	Inflammation (BALF & Histology) Lung particle burden Cytotoxicity (LDH) & permeability (protein)	High concentrations of particles administered impaired their clearance from the lung. However, hamsters were able to most efficiently clear particles. Inflammatory response evident in all species, but was most severe and persistent in rats. Increased LDH & protein (least severe in hamsters) Species differences, and dose dependent effects observed

Bermudez et al., [7]	TiO_2 _(21 nm)	Inhalation (13 week exposure) -mice, rats, hamsters	Inflammation (BALF & Histology) Lung particle burden Cytotoxicity (LDH) & permeability (protein)	Retained particle burden decreased with (post-exposure) time & particles contained in macrophages Increased cellular infiltration (macrophages and neutrophils) dependent on species Increased LDH & protein (not hamsters) • Findings dependent on species (rats>mice>hamsters) and particle concentration

Heinrich et al., [13]	TiO_2_ (also diesel soot and ufCB treatment groups)	Inhalation (2 year exposure (with satalite groups at 3, 6, 12 & 18 months), with or without subsequent clean air exposure for 6 months post particle exposure) -rats and mice	Histology (to assess Carcinogenicity) DNA adducts Lung particle burden Alveolar lung clearance BALF cytology and biochemical (including LDH, protein) analysis	Increased mortality with TiO_2 _(although mortality was also high in the control group) Alveolar lung clearance compromised by TiO_2_ Increased protein, LDH in BALF Increased lung tumours

Ferin et al., [3]	TiO_2 _(12, 21, 230 & 250 nm)	Intratracheal instillation (24 hour exposure) Inhalation (12 week exposure) -rats	Inflammation (BALF neutrophil infiltration & histology) Lung burden & particle clearance	Neutrophil infiltration (greater for smaller particles) Particles internalised by alveolar macrophages Particle clearance slower for smaller particles, and access the pulmonary interstitium to a larger extent than fine particles

Grassian et al., [10]	TiO_2 _(5 & 21 nm)	Inhalation (4 hour exposure will observations made immediately or 24 hours post exposure) Nasal instillation (24 hour exposure) -mice	Inflammation (BALF cells & cytokines) Cytotoxicity (BALF LDH) Lung Permeability (BALF protein) Lung histopathology (inflammation, lung injury, and abnormalities in pulmonary architecture)	Inhalation: macrophage infiltration, no changes in protein, LDH & histopathology Nasal instillation: neutrophil infiltration, increased IL-1β, IL-6, protein and LDH for 21 nm NPs only • 21 nm NPs more toxic than 5 nm NPs (due to agglomeration differences)

Warheit et al., [9]	6 samples of TiO_2 _(of various surface coatings, size up to 440 nm)	Inhalation (4 week exposure, with observations made at 2 weeks to 1 year post exposure) Intratracheal instillation (24 hours to 3 month exposure) -rats	Inflammation (BALF) Lung permeability (BALF protein) Cytotoxicity (BALF LDH) Histopathology	Inhalation: particle containing macrophage accumulation, epithelial cell hyperplasia, fibrotic response (collagen deposition) Intratracheal: neutrophil infiltration, particle laden macrophages, increased lung permeability, ncreased cytotoxicity • Surface treatment paramount to toxicity: aluminia and silica coatings increase toxic potency • The pulmonary toxicity of the particle panel overall was low & was similar in inhalation and instillation set ups

As mentioned previously, a study conducted by Ferin et al. [3] was one of the first to demonstrate that particle size was fundamental to the pulmonary toxicity of TiO_2_. In this study, rats were exposed via inhalation, to 21 nm (23.5 mg/m^3^) and 250 nm (23.0 mg/m^3^) TiO_2 _for 12 weeks, and examination of the consequences of TiO_2 _exposure evaluated over a 64 week post-exposure period. Alternatively, rats were administered TiO_2 _of various sizes (12, 21, 230 and 250 nm in diameter) via a single intratracheal instillation (up to 1000 μg/rat) and toxicological investigations made 24 hours post-exposure. Following intratracheal or inhalation exposure, nanoparticulate TiO_2 _induced a greater pulmonary inflammatory response (characterised by neutrophil infiltration), than its microparticulate (250 nm) counterpart, which did not elicit any changes in the inflammatory status of the lung. The smaller sized TiO_2 _particles were also found to remain within the lung for longer periods (501 days) following inhalation, than the larger TiO_2 _particles (174 days). This demonstrates that the clearance of smaller particles from the lung was slower. In fact, the prolonged retention of smaller TiO_2_particles in the lung was suggested to derive from the finding that they were able to translocate to the pulmonary interstitium more efficiently than the larger TiO_2 _particles. The authors proposed that this phenomenon was facilitated by the fact that smaller particles were not efficiently taken up by alveolar macrophages, which thereby allowed for their prolonged interaction with alveolar epithelial cells. It was found that an increased mass dose (which translates to an increased number of particles, for smaller particles) promoted the movement of particles within the pulmonary system. It was therefore observed that particle size, and the number of particles administered (which was related to the delivered dose) impacted on the translocation process, and therefore TiO_2 _toxicity. However, since the publication of this study it has been demonstrated that the surface area of particles is influential to their toxicity. As such, smaller particles have a greater surface area than their larger counterparts, and as a consequence exhibit greater toxicity, when administered at an equal mass dose.

The size dependency of TiO_2 _(and carbon black (CB)) toxicity was investigated by Renwick et al. [6]. To achieve this, particle mediated inflammatory responses were investigated, and the ability of the particles to impact on clearance mechanisms within the rat lung determined. Rats were exposed to particles via intratracheal instillation, at 125 μg/rat or the relatively high dose of 500 μg/rat, and toxicological investigations conducted at 24 hours post exposure. At the highest dose only, nanoparticulate (29 nm), but not microparticulate (250 nm) TiO_2 _stimulated the recruitment of neutrophils into the lungs, epithelial damage, increased permeability of the lung epithelium, and cytotoxicity, which were measured within the bronchoalveolar lavage fluid (BALF). The NPs were also able to diminish the phagocytic ability of isolated rat alveolar macrophages (from exposed animals), and increase the ability of these macrophages to migrate towards the chemotactic signal, C5a. The authors suggested that the consequences of such a stimulation would be to promote the retention of macrophages, and therefore their particle burden, within the lung. The NPs were therefore demonstrated to elicit a greater pulmonary inflammatory response than their larger counterparts.

In a different approach, Bermudez et al. [7] determined if the choice of species was able to influence the pulmonary response to nanoparticulate TiO_2 _(21 nm). This was achieved through the exposure of rats, mice and hamsters, to TiO_2 _NPs, via inhalation, for 13 weeks (6 hours/day, 5 days/week) at concentrations of 0.5, 2, or 10 mg/m^3^. The pulmonary response was assessed up to 52 weeks post exposure, and included assessment of inflammation, cytotoxicity, lung cell proliferation and histopathological analysis. It was demonstrated that a pulmonary inflammatory response was stimulated by TiO_2 _within mice and rats, but was absent in hamsters. The nature of the response was also observed to vary within the different species. Specifically, a greater neutrophillic response, which decreased with time, was apparent in rats, with progressive epithelial fibroproliferative changes also apparent. In mice, there was neutrophil and macrophage component of the inflammatory response, and these cell populations remained elevated throughout the observation time. The severity of the response was ranked in the following order, by the authors; rat>mouse>hamster. Consequently, the rat was found to be the most sensitive species to the effects of TiO_2_. The limited toxicity apparent within hamsters was thought to derive from the low lung burden of particles, as hamsters had the greatest propensity to efficiently clear particles from the lung. However, for mice and rats, the initial retention of particles was similar, and decreased with time, demonstrating that the pulmonary clearance kinetics varied amongst the different species. The study highlights that differences in the response, and therefore sensitivity of different species are worthy of consideration within investigations, and the model which mimics the human situation most accurately requires assessment. It is also of note that the effects observed were dose dependent, and so the toxicity of NPs was enhanced by increasing the concentration. The findings demonstrated within this study were comparable with those of a companion study conducted by Bermudez et al. [8]. In this study rats, mice and hamsters were exposed to fine TiO_2 _particles (1 μm), via inhalation, at concentrations of 10, 50 or 250 mg/m^3^, with the experimental design identical to that described previously [7]. Again, species differences were witnessed, so that some species were most susceptible to particle exposure (rat>mouse>hamster), in addition to evidence that the pulmonary toxicity observed was concentration dependent. However, it is difficult to distinguish if size dependent effects were observed within these separate studies. This is derived from the utilisation of a higher dose of particles within the microparticulate study, when compared to those encountered within the NP study. However, as no effects were elicited by fine TiO_2 _particles when administered at a concentration of 10 mg/m^3 ^it is reasonable to assume that the NP sample is more toxic, when administered at the same mass dose, as toxic effects were observed at this dose for NPs. In fact, the concentration of particles used were exceptionally high in both studies, and thus their physiological relevance is questionable (see later). However, the authors aimed to achieve a particle overload situation (see later), so that the administration of high particle concentrations was undertaken to investigate the efficiency of clearance processes in the different species, and thus the experimental set up can therefore be justified.

The ability of the experimental set up to influence the pulmonary response to TiO_2 _was also investigated by Warheit et al. [9]. It was determined if the exposure method, or TiO_2 _formulation (specifically concentrating on surface coating modification) were able to impact on their pulmonary toxicity within rats. For inhalation, rats were exposed to particles (ranging from 290 to 440 nm in diameter) at concentrations of up to 1300 mg/m^3^, for a duration of 4 weeks. In general, following inhalation of TiO_2_, there was an accumulation of particle containing macrophages within the lungs. Following intratracheal instillation (up to 10 mg/kg) it was observed that some TiO_2 _preparations were able to stimulate a transient, pulmonary inflammatory response that was typified by the infiltration of neutrophils, and lactate dehydrogenase (LDH) release, and that this response was resolved within one week post-exposure. It is relevant that the response that transpired following particle exposure was very much dependent on the TiO_2 _formulation in question. Specifically, the samples that contained the highest alumina or amorphous silica content elicited the greatest adverse pulmonary response. Overall, both exposure methods were associated with minimal adverse effects, but this may be derived from the size of particles utilised, as all samples were out-with nano dimensions (i.e. >100 nm). It must also be noted that the concentrations of particles administered in this experiment are deemed to be excessive, and therefore the relevancy of the findings are questionable. The utilisation of high particle concentrations is a common problem within this field of research, as they are not expected to be encountered within humans, and thus this issue will be discussed in greater detail later.

Similarly, Grassian et al. [10] investigated what particle attributes drove the toxicity of TiO_2 _NPs (5 and 21 nm) within mice, subsequent to inhalation (0.7 or 7 mg/m^3^, for 4 hours with toxicological observations made immediately and 24 hours post exposure) or nasal instillation (up to 12.7 μg/mouse). An elevated macrophage population was associated with the inhalation of particles, and these cells were observed to internalise particles. An infiltration of neutrophils, was associated with the nasal instillation of TiO_2_. In both exposure scenarios, the response induced by 21 nm particles was greater than that exerted by their 5 nm counterparts. Consequently, the surface area of particles was not deemed to be the sole determinant of TiO_2 _toxicity, which was unexpected. Instead, the crystallinity of the samples was suggested to influence the toxicity of TiO_2 _(see later), as 5 nm particles were pure anatase, whereas the 21 nm particles were a mixture of rutile and anatase forms of TiO_2_. It is also relevant that the nature of the response varied for the different exposure methods, specifically inhalation exposure promoted a macrophage driven response, and instillation exposure triggered the development of a neutrophil driven response. This insinuates that the experimental set up influences the results of toxicity tests, and may be related to differences within their distribution within the lung following exposure, which requires further investigation.

In line with these findings, Warheit et al. [11] aimed to determine if the crystalline form of TiO_2 _was able to influence its pulmonary toxicity within rats, subsequent to intratracheal administration (1 or 5 mg/kg). Two rutile NP samples, a 'mixed' NP (80% anatase, 20% rutile) sample, and a microparticulate rutile (negative particulate control), in addition to α-quartz sample (included as a positive particulate control) were considered. All TiO_2 _particle samples were highly agglomerated (>2000 nm), following their dispersion in phosphate buffered saline (PBS), despite the fact that their primary particle size was less than 100 nm. The pulmonary response to the particle panel was evaluated up to 3 months post exposure. Although quartz had the greatest inflammogenicity, it was evident that several TiO_2 _particle types elicited a short-term, pulmonary inflammatory response that was characterised by the infiltration of neutrophils, and was greatest, and more sustained for the mixed NP sample. Furthermore the only samples capable of eliciting a release of LDH (a measure of cytotoxicity) and protein (indicative of increased vascular permeability) into BALF were quartz and the mixed TiO_2 _sample. The properties responsible for driving TiO_2 _toxicity were then considered and it was suggested that differences in particle surface area accounted, in part, for the responses exhibited by particles. Accordingly, the mixed sample had the greatest surface area (53 m^2^/g), and therefore greatest reactivity, so that surface properties were suggested to drive TiO_2 _toxicity. However as the rutile NP samples had surface areas of 18.2 m^2^/g and 35.7 m^2^/g, and the TiO_2 _microparticles had a surface area of 5.6 m^2^/g, it was expected that the rutile NP particle samples would exhibit an intermediate level of toxicity (between the mixed, and microparticulate samples), but this did not transpire within the findings. Therefore, additional factors were speculated to contribute to the toxicity of TiO_2 _particulates, besides their surface area, such as crystallinity. It was suggested that the rutile NP samples were less toxic than those that were predominantly anatase (see later). In addition, the authors suggested that post production processing, that removed chloride from the particle surface, and enhanced agglomeration of particles, may also contribute to the reduced toxicity of the rutile NP particles. Again, the particle concentrations administered were excessively high, and unlikely to be encountered by humans, and therefore justification of such concentrations is required by investigators, in order to determine their relevancy.

The nature of the pathological response associated with the pulmonary exposure to TiO_2 _has been studied by a number of investigators. Chen et al. [12] exposed mice, via intratracheal instillation (0.1 and 0.5 mg/mouse), to nano (19-21 nm) and micro (180-250 nm) forms of TiO_2_. Pulmonary toxicity was determined 3 days, 1 week or 2 weeks post exposure. Histological assessment illustrated that morphological alterations within the lung were evident on exposure to nanoTiO_2_, which were emphysema-like in nature (including, for example alveolar enlargement). Lesions were more pronounced in areas where particles preferentially accumulated, and increased in severity, with increasing time and dose. An inflammatory response, recognised by the infiltration of macrophages (that were particle laden), upregulation of cytokines (including monocyte chemotactic protein (MCP)-1, interleukin (IL)-1, tumour necrosis alpha (TNFα) and several neutrophil chemoattractants) and complement activation was also observed. Despite the large doses used, no pathological lesions were found in response to microparticulate TiO_2_. This study again illustrates the size dependent toxicity exhibited by TiO_2_, and the tissue lesions, and biochemical markers associated with the particle toxicity.

In a different approach, Heinrich et al. [13] exposed rats to P25 TiO_2 _NPs (size reported by others to be 21 nm (see for example Ferin et al [3]) via inhalation for 2 years. Particle exposure was followed by exposure to clean air for 6 months. In addition, mice were exposed to TiO_2 _for 13.5 months then clean air for 9.5 months. TiO_2 _was able to increase mortality within rats and mice, so that their lifetime was significantly shortened. Lung tumours were observed in both species, as a consequence of the chronic exposure to TiO_2_. The high doses utilised in this experiment undermines the relevancy of the findings In fact, they are likely to represent particle 'overload' conditions, where the lung is overwhelmed by particle presence to enhance the toxicity that is manifested. Overload can be described as the deposition of particles that exceeds the ability of macrophages to remove them. As a consequence, particles are more likely to be retained within the lung, and interact with the different lung cell populations to stimulate toxicity, such as tumour development. This study therefore highlights how the experimental set up has the ability to influence the findings (see later). Consequently, TiO_2 _cannot be regarded as carcinogenic based on the findings from this study. Therefore, it is anticipated that the administration of excessively high concentrations of particles was responsible for promoting tumour development within this model, and not an inherent genotoxic property exhibited by TiO_2 _particles.

Ahn et al. [14] investigated what processes were responsible for particulate mediated stimulation of excessive mucus secretion within humans. To achieve this, TiO_2 _(4 mg/kg, 0.29 μm diameter) was exposed to rats via intratracheal instillation, and toxicological observations made from 4 to 72 hours post exposure. TiO_2 _exposure stimulated an increase in goblet cell hyperplasia, which is, in part, attributed to an increase in muc5 gene expression and IL-13 production. Therefore, it could be speculated that particle mediated increases in mucus secretion can contribute to the aggravation of chronic airway disease symptoms within humans, and therefore warrants further investigation.

The ability of TiO_2 _exposure to influence the pulmonary toxicity of known inflammogenic agents has been investigated by Inoue et al. [15]. The impact of TiO_2 _(15, 50, 100 nm) intratracheal instillation (8 mg/kg) on lipopolysaccharide (LPS) mediated pulmonary inflammation in mice was determined. Combined TiO_2 _and LPS treatment was able to exacerbate LPS mediated inflammation (indicated by keratinocyte chemoattractant (KC), IL-1β and MCP-1 production). Circulating cytokines were also increased, as well as coagulatory factors such as fibrinogen. As observed previously, there was a size dependency to the findings was also observed, whereby smaller particles were most toxic.

In a different approach, Nurkiewicz et al. [16] determined the impact of TiO_2 _particles (1 μm) or ROFA particulate matter on the systemic microvasculature, following intratracheal instillation of rats (0.1 or 0.25 mg/rat, for 24 hours). The authors therefore encompassed the possibility that systemic effects were a component of the pulmonary response to particulates. Particle exposure stimulated a neutrophil influx into the lungs, but no cytotoxic response was evident. Particles were able to induce an impairment of endothelium dependent arteriolar dilation. The response was suggested to be related to increased neutrophil adhesion to the vessels, myleoperoxidase (MPO) deposition, and oxidative stress within the vessel wall. These findings are of concern, as they indicate that an inflammatory response may be stimulated within the vessel. However, the response was independent of the level of pulmonary inflammation, and was not thought to be reliant on the migration of particles from the lung. This study highlighted the systemic (inflammatory mediated) responses that are associated with particle exposure, but at this time the mechanisms underlying this response is unknown.

Consideration of the fate of TiO_2_, subsequent to pulmonary exposure has been apparent within a number of investigations, with a particular focus on the transfer of particles to the brain. Wang et al. [17] investigated the distribution of rutile (80 nm) and anatase (155 nm) TiO_2 _particles within the mouse brain, following nasal instillation exposure (500 μg/mouse, every other day, for a total of 30 days) and determined if any neurotoxicity was associated with exposure. Both forms of TiO_2 _were able to access the brain, with accumulation within the cerebral cortex, thalamus and hippocampus evident, and postulated to occur via the olfactory bulb. This route of uptake however, was unlikely to be mediated via penetration into the cardiovascular system and via the blood. Instead, TiO_2 _delivery to the brain was speculated to occur via neuronal transport. Accumulation of TiO_2 _resulted in morphological alterations and loss of neurones in the hippocampus, which was accounted for by the higher distribution of TiO_2 _within this brain region. In addition, it was suggested that TiO_2 _elicited oxidative stress within the brain due to elevated catalase activity, decreased superoxide dismutase (SOD) activity and evidence of increased lipid peroxidation and protein oxidation. Therefore neuronal mediated translocation of TiO_2 _to the brain, following nasal instillation, was observed, with the hippocampus illustrated as being the main target of accumulation and toxicity. Wang et al. [18] expanded upon these findings and found that the phenomenon was time dependent (maximal at 30 days), and that an inflammatory response (indicated by IL-1β, and TNFα) within the brain was also stimulated by TiO_2 _exposure. The response was measured at day 2, 10, 20, and 30. It was apparent that repeated exposures, over a period of 30 days, were required to enable the accumulation of TiO_2 _within the brain. It is therefore of interest that the neuronal transport of NP containing substances between the nose and CNS could be exploited, in order to bypass the blood brain barrier, but that the stimulation of an inflammatory response may be associated with this phenomenon.

The results of the outlined studies imply that the toxic potential of TiO_2 _was primarily dictated by particle size, and crystallinity; whereby decreasing particle size, and anatase forms of TiO_2 _were observed to enhance particle pulmonary toxicity. In addition, it is suggested that the experimental model (including species used, exposure method and dose administered) was able to impact on the toxicity of TiO_2_, which complicates making comparisons between different investigations. The pulmonary response to TiO_2 _has been demonstrated to be inflammogenic; with a contribution from neutrophils and macrophages (which are elevated and thought to contribute to particle removal). In addition, epithelial damage, oxidative stress and cytotoxicity are also common findings. In addition, chronic exposure to TiO_2 _also has the ability to promote tumour development, illustrating their carcinogenic potential, but this is only likely to manifest when administered in particle overload conditions.

## Intraperitoneal exposure to TiO_2_

Chen et al. [19] investigated the acute toxicity of nano-TiO_2 _(80-100 nm) subsequent to the intraperitoneal injection of mice. The doses used were exceptionally high (ranging from 324 to 2592 mg/kg), and it is therefore unsurprising that mortality was associated with exposure. TiO_2 _was observed to block pulmonary vessels, leading to thrombosis, with pathology also evident within the liver, spleen and kidneys. This study highlighted the need to consider the use of realistic doses of particles, as the observed effects are likely to derive from the high dose of particles administered, and not the inherent toxicity of particles.

## Dermal exposure to TiO_2_

Considering the impact of TiO_2 _dermal exposure is especially relevant due to its inclusion within sunscreens and cosmetics that are directly applied to skin. As a consequence, the dermal penetration and toxicity of TiO_2 _particles, has been a focus of a number of investigations. The skin, and in particular, the stratum corneum, is a primary barrier against the penetration of particles contained within dermally exposed substances, and therefore its efficiency will also be discussed.

Mavon et al. [20] determined the distribution of TiO_2 _(20 nm) within the skin *in vitro *and *in vivo*. Five hours following the direct topical application of TiO_2 _(2 mg/cm^2^) to human skin or to human skin explants tape stripping (placing and removing adhesive tape onto and from the skin repeatedly and subsequently analysing the components striped from the skin surface) was used to determine the dermal penetration of TiO_2_. *In vivo*, and *in vitro*, the majority of the TiO_2 _was contained within the stratum corneum, with minimal distribution within the epidermis. Therefore in both preparations, TiO_2 _presence decreased with increasing depth of the skin, so that the penetration of TiO_2 _past the stratum corneum into viable skin layers was minimal.

Similarly, Schulz et al. [21] determined the influence of particle size, coating and shape on TiO_2 _skin penetration. A number of TiO_2 _containing sunscreen formulations were tested that had different particle surface characteristics; trimethyloctylsilane coated (20 nm), or aluminium oxide (Al_2_O_3_) and silica (SiO_2_) coated (10-15 nm). Formulations were topically exposed to human skin at a concentration of 4 mg/cm^2^, for 6 hours, and skin biopsies taken. All particle types were solely located on the outermost surface of the stratum corneum, and did not penetrate deeper to subcutaneous, epidermis or dermis layers.

In a different approach, Kiss et al. [22] evaluated the barrier function of skin, within human foreskin grafts transplanted onto severe immunodeficient mice. A commercially available TiO_2 _microparticulate containing sunscreen was administered (2 mg/cm^2^) via an occlusive bandage to skin grafts for 24 hours, and the penetration of TiO_2 _determined within skin biopsies. It was found that TiO_2 _particles did not penetrate through the stratum corneum of human skin transplants. The stratum corneum was therefore deemed to be an adequate, effective barrier against TiO_2 _penetration in intact human skin. However, the authors noted that as it is likely that TiO_2 _exposure occurs when skin barrier functions may be impaired (such as sunburn), and as a consequence, TiO_2 _may come into direct contact with underlying skin cells, a concept which has yet to be investigated.

The findings from the available studies demonstrated that the penetration of TiO_2 _is negligible within (healthy) skin. This is important, as NPs appear to be unable to reach the living cells present within the deeper skin layers, and thus their propensity for toxicity is anticipated to be minimal. Therefore, it is relevant, within future studies, to consider the fate of particles within skin models that take into consideration that sunscreens are often applied to burnt, damaged and diseased skin, where the barrier function of the stratum corneum will inevitably be impaired [22,23]. This has been discussed within several investigations but not actually studied.

## Oral exposure to TiO_2_

Only one study could be identified that addressed the consequences of oral exposure of TiO_2_. Wang et al. [24] investigated the distribution and acute toxicity of nanoparticulate (25 and 80 nm) and microparticulate (155 nm) forms of TiO_2 _in mice, following oral exposure (5 g/kg). TiO_2 _particles of all sizes, 2 weeks post exposure, were distributed to the liver, spleen, lungs, kidneys, thus providing evidence that they could be transported to other sites subsequent to exposure, due to their translocation into the blood. Within the liver, NPs initiated an inflammatory response, with liver damage indicated by a rise in serum transanimases, and hepatic necrosis was revealed in histopathological investigations. Markers of cardiac damage were also observed to be elevated by TiO_2 _NPs, within the serum. Limited toxicity was associated with microparticle exposure. This study therefore provided interesting results. Specifically the potential for TiO_2 _particle transfer into the blood from the GIT was demonstrated, which would necessitate that they are able to pass through the gut wall. It also revealed that the liver was a primary target for the toxicity of TiO_2_. However, the use of an exceptionally high dose of particles undermines the relevancy of the results, as such exposure levels are unexpected in humans.

## ADME Profile of TiO_2_

Determining the kinetics of TiO_2 _within the body, subsequent to exposure (via the lungs, gut and skin) is necessary to identify potential targets of toxicity. The delivery of TiO_2 _particles from the exposure site to secondary target organs, such as the liver or brain requires their transfer into blood, or transport within neurones. Therefore their likelihood of accessing different organs and tissues within the body is also necessary to direct appropriate *in vitro *investigations at relevant target sites. However, a number of barriers (at the exposure site and those apparent within secondary targets) are in place to prevent against uptake, and it is necessary to determine if they are overcome by TiO_2_, to determine its systemic availability.

Only one example of particle translocation into the blood from the gut could be identified. Specifically, TiO_2 _NPs were observed to translocate into the blood, following oral exposure, and thereafter distribute to secondary targets, including the liver, spleen, lungs, and kidneys [24]. Studies investigating the dermal penetration of TiO_2 _suggest that the transport of nanoparticles past the stratum corneum is negligible, and therefore it is unlikely that the particles will access the circulation via this route. The limited penetration of TiO_2 _NPs within skin has also been replicated within *in vitro *models (see later). Wang *et al*. [17,18] demonstrated the transfer of TiO_2 _NPs to the brain, following intranasal exposure, which occurred via their transport within neurones.

No evidence of TiO_2 _metabolism, or elimination from the body could be identified in the literature at this time.

The translocation of TiO_2 _particles, subsequent to pulmonary and oral exposure should encompass the possibility that distal sites are affected; including the CNS, liver, and cardiovascular system. The translocation is likely to be due to their passage into blood, but may also be mediated by neuronal transport. Both these processes would allow for their transport to targets sites (such the brain), and within different regions of a particular target (such as different brain regions). In contrast, the penetration of particles within the skin is negligible, and so particles are unlikely to become systemically available, following exposure via this route. The elimination of particles from the body is also a necessary consideration, which would provide information regarding the longevity of particles within the body, and therefore their propensity for damage.

## Distribution of TiO_2 _following intravenous exposure

Following injection, it is necessary to determine particle distribution to identify potential targets for toxicity. There are limited available studies that purport to investigate this. In addition, it is assumed that if NPs gain access to the circulation following exposure via the lungs, skin or GIT, a similar distribution pattern is expected, albeit it to a lesser extent.

Fabian et al. [25] determined the tissue distribution of TiO_2 _NPs (20-30 nm) within rats following intravenous injection (5 mg/kg), 1, 14 and 28 days post exposure, TiO_2 _was cleared from the blood and primarily accumulated within the liver, but was also apparent within the spleen, lungs and kidneys. The level of TiO_2 _within the liver was maintained over the observation time, however levels decreased with time within the other organs. No serum cytokine or enzyme changes were observed, which insinuated that no toxicity was associated with TiO_2 _exposure, however further investigations, including histopathological analysis would be necessary to confirm this.

The only study identified suggested that following intravenous administration, TiO_2 _particles are cleared from the blood and are able to accumulate primarily within the liver, but also the lungs and spleen. This is likely to derive from their uptake by the resident macrophage populations, contained within the reticulendothelial system. Further investigations are necessary to determine if an inflammatory or toxic response is associated with such an accumulation.

## *In vitro *investigations of TiO_2 _toxicity

### Lung models

Investigations that assess the pulmonary toxicity of TiO_2 _particles *in vitro *have used a variety of models including macrophages (due to their contribution to clearance), epithelial cells (due to their abundance, and expected interaction with particles) and explanted tissue.

Park et al. [26] investigated the cytotoxicity of TiO_2 _NPs (21 nm) to BEAS-2B lung epithelial cells, at concentrations ranging from 5 to 40 μg/ml, for up to 96 hours. A dose and time dependent decrease in cell viability was observed. Caspase-3 was activated by TiO_2_, with chromosome condensation also apparent, which was suggestive that an apoptotic mechanism of cell death was involved. Reactive oxygen species (ROS) production, glutathione (GSH) depletion, and increased hemeoxygenase (HO-1) expression was evident, thereby implying that an oxidative mechanism drove the cytotoxic response. IL-8, IL-1, IL-6, TNFα, and CXCL2 (neutrophil chemoattractant) cytokine gene expression was increased, insinuating that an inflammatory response was also stimulated by TiO_2_. The findings therefore indicated that an oxidant driven response was integral to the toxicity of TiO_2_, which had inflammatory and cytotoxic consequences.

Churg et al. [27] determined the impact of TiO_2 _exposure (up to 7 days), on the development of a fibrotic response, within the airway wall on rat tracheal explants. Both microparticulate (0.12 μm) and nanoparticulate (0.021 μm) forms of TiO_2 _were investigated. No changes in gene expression were observed until day 5. NPs stimulated growth factor expression (such as platelet-derived growth factor (PDGF)) and enhanced pro-collagen expression. The authors suggested that the NPs were more fibrogenic, which is anticipated to contribute to airway obstruction *in vivo*. However, TGFα and TGFβ expression was increased for microparticles only, but no fibrotic-like morphological changes occurred, and so it was suggested that these mediators were not responsible for driving the fibrotic response mediated by NPs.

Gurr et al. [28] investigated the oxidative damage exhibited by TiO_2 _(10, 20 or >200 nm) within bronchial BEAS-2B cells. The oxidative potential of TiO_2 _was confirmed by the finding that lipid peroxidation (indicated by increased malondialdehyde (MDA)) and ROS (hydrogen peroxide) and reactive nitrogen species (NO^.^) production were enhanced by nanoparticulate forms of TiO_2_. DNA adducts were only formed following exposure of cells to TiO_2 _NPs. The oxidative and genotoxic potential of nanoparticulate forms of TiO_2 _was therefore demonstrated to be superior to that of their larger counterparts.

Simon-Deckers et al. [29] investigated the toxicity of a panel of particles, including TiO_2 _(in anatase and rutile forms, and a variety of sizes) and Al_2_O_3_, to A549 cells (a human carcinoma derived alveolar epithelial type 2 cell line). In general, the cytotoxicity of the particles was low, despite the fact that they were internalised by cells, and contained within cytoplasmic vacuoles or lysosomes. However, it is noteworthy that it is possible for particles to be internalised by cells without having a detrimental impact on normal cell function. The crystal phase of TiO_2 _was observed to influence the cytotoxicity exhibited by particles. Specifically, the greater the anatase content of the sample, the greater the ability to induce cell death. In addition, the size of NPs was suggested to contribute to their toxicity, as TiO_2 _NPs were more toxic than their larger counterparts. In addition, the chemical composition of NPs was also thought to impact on their toxicity, as 12 nm TiO_2 _NPs were more toxic than 11 nm Al_2_O_3_, despite their similarity in size. Consequently, the size, composition and crystal phase of particles were all suggested to influence NP toxicity, despite the fact that the toxicity of NPs used was generally low. Therefore, it is acknowledged that investigating size dependent effects of particles is confounded by differences in other particles attributes such as the sample phase. Consequently determining the properties of TiO_2 _that drive toxicity is difficult, and therefore isolating a particle characteristic that is solely responsible for mediating adverse effects is not likely to be attainable.

Kim et al. [30] assessed TiO_2 _(1 μm) mediated cytotoxicity to alveolar macrophages (obtained from rats, via bronchoalveolar lavage), when exposed to concentrations ranging from 0.5 to 5 mg/ml, for up to 5 hours. TiO_2 _elicited a dose dependent decrease in cell viability, which was suggested to be mediated by suppressed adenosine triphosphate (ATP) generation, and also reliant on the interaction of TiO_2 _with scavenger receptors on the cell surface. It is relevant that the response of silica particles (1.6 μm) was also considered. Silica had a greater cytotoxic potential than TiO_2 _and the mechanisms underlying the response were different.

As responses of organs and tissues are likely to involve interactions between different cell types, it is necessary to incorporate this within *in vitro *tests. Barlow et al. [31] investigated the ability of L2 epithelial cells to modulate macrophage migration *in vitro*, on exposure to nano and microparticulate forms of TiO_2 _(29 nm and 250 nm, respectively) and CB (260 nm and 14 nm diameter respectively). TiO_2 _and CB induced a dose dependent decrease in epithelial cell viability, which was greatest for NPs (when exposed at concentrations ranging from 62.5 to 2000 μg/ml for 24 hours). Conditioned medium (obtained from epithelial cells treated with particles) was able to increase macrophage migration, but only for ultrafine carbon black NPs. Therefore, TiO_2 _NPs were unable to stimulate the release of chemotaxins from epithelial cells, which the authors suggest could be due to their smaller surface area, when compared to that of ufCB.

From the investigations discussed, it is evident that TiO_2 _particles are able to detrimentally affect both lung derived macrophage and epithelial cells. Therefore, in general, particles mediated oxidative, inflammatory and genotoxic effects that eventually culminated in cytotoxicity. However, in some situations there was no evidence of toxicity, mediated by TiO_2_. These differences may be derived from the model used, or the particle under investigation. It is of interest that the mechanisms driving the toxicity of TiO_2 _*in vitro *(particularly inflammation and oxidative stress) are also witnessed *in vivo *(see earlier).

### Dermal models

The penetration of TiO_2 _particles within the skin has been a focus of a number of *in vitro *investigations due to the exploitation of particles within sunscreens and cosmetic products. Gamer et al. [32] evaluated the penetration of sunscreen formulations containing micron-sized TiO_2 _(up to 400 μg/cm^2^, for 3 to 24 hours) within porcine skin explants. It was observed that the location of both particle types was restricted to the stratum corneum following their topical application. Specifically, particles were deposited on the skin surface, within the outmost layer of the stratum corneum, as evidenced by their removal via washing or tape stripping analysis. There was therefore no evidence that particles penetrated into the deeper stratum corneum layers, epidermis or dermis, and thus the barrier function of the skin was successful in limiting the passage of particles.

Similarly, Pflucker et al. [33] illustrated that TiO_2 _NPs (20-50 nm) did not penetrate porcine skin *in vitro*. A TiO_2 _emulsion (4 mg/cm^2^) was topically applied to excised pig skin for 24 hours. Skin biopsies were taken following exposure, and the penetration of particles assessed using tape stripping. Again TiO_2 _was exclusively located within the outermost layer of the stratum corneum, with no distribution to the underlying living cell layers evident. Consequently, this study suggests that the intact stratum corneum is an effective barrier to TiO_2_, and that the penetration of particles within the skin is negligible.

In addition, Dussert et al. [34] investigated the distribution of TiO_2 _and ZnO NP containing sunscreen formulations within human skin explants. Again, sunscreens were topically applied to skin but no penetration of particles, or intracellular delivery was observed past the stratum corneum skin surface.

The functional implications of the exposure of the various skin cell populations have also been determined within *in vitro *investigations. From the data available for TiO_2 _particles, such studies are potentially more relevant to compromised/damaged/diseased skin than normal healthy skin, due to the negligible penetration of particles within normal skin. There is therefore a need to consider the distribution of particles within damaged or sunburnt skin, which would be likely to promote the penetration of particles, due to damage to the stratum corneum. There is little or no data currently available to assess the impact of such damage on penetration or hazard.

Kiss et al. [22] exposed HaCaT keratinocytes, human dermal fibroblast cells, SZ95 sebaceous gland cells and primary human melanocytes to TiO_2 _(9 nm diameter), at concentrations of 0.15 to 15 μg/cm^2^, for up to 4 days. Particles were internalised, and evident in the cytoplasm and perinuclear region of fibroblasts and meloncytes. Particle uptake was also associated within an increase in intracellular calcium in these cell types. However, no particle uptake, or alterations in calcium signalling were observed within keratinocytes or sebocytes. A dose and time dependent decrease in cell proliferation was evident within all cell types, and an increase in cell death (via apoptosis) within fibroblasts was apparent. The direct contact of cells with TiO_2 _particles is therefore of concern, as it can disturb normal cell functions within the different skin cell populations, but it is noteworthy that all cell types were not affected similarly, and thus cell specific effects are encountered on exposure to TiO_2 _particles. Kiss et al. [22] also demonstrated that the stratum corneum was an effective barrier against micronized TiO_2 _*in vivo *(see earlier). However it would have been of greater interest to determine if NPs are able to penetrate skin, as the *in vitro *component of the study illustrated that when in direct contact with skin cells TiO_2 _NPs are able to elicit toxicity.

Jin et al. [35] determined the cytotoxicity of TiO_2 _(20-100 nm) to mouse L929 fibroblasts at concentrations of 3-600 μg/ml, for up to 48 hours. There was a time and dose dependent decrease in cell viability induced by TiO_2_. An increase in ROS production and decreases in GSH and SOD activity were observed, thereby implying that oxidative stress was a feature of the response. TiO_2 _was also demonstrated to be internalised by phagocytosis, and was contained within lysosomes. Analysis of cell morphology illustrated that cell morphology was detrimentally affected by TiO_2_, and that cell adhesion and proliferation was prevented, confirming the decrease in cell survival.

The studies conducted demonstrated that the toxicity of TiO_2 _to skin cells is of concern, with oxidative, genotoxic and cytotoxic consequences evident, and likely to act in concert. However, in order to execute these effects, particles would be required to penetrate past the stratum corneum, to reach the underlying cell populations, which is unlikely to occur in healthy skin (see earlier). As noted previously, the penetration of particles within damaged skin should therefore be considered a priority in future experiments.

### GIT models

Only one study could be identified that evaluated the impact of TiO_2 _exposure on the GIT, *in vitro*. Zhang et al. [36] investigated the contribution of photoexcitation, to the cytotoxicity of TiO_2 _(21 nm) within human colon carcinoma Ls-174-t cells. Cells were exposed to TiO_2 _for 24 hours, at concentrations up to 1000 μg/ml. Limited cytotoxicity of cells was observed, following TiO_2 _exposure, in the absence of ultraviolet (UV)A irradiation. In contrast, a high level of TiO_2 _induced cell death was observed in the presence of UVA light, which was a dose and time dependent phenomenon. Therefore, the toxicity of TiO_2 _had a photocatalytic component. Consequently, TiO_2 _particles and light irradiation therapy was suggested by the authors to be a suitable candidate for the treatment of cancer, and warrants further investigation. However, this may be limited by the ability of UV light to penetrate the human body, and the specificity of the response, and so the exploitation of this for tumour treatment will inevitably be restricted.

### Liver models

Hussain et al. [37] compared the impact of a range of NPs, including iron oxide (Fe_3_O_4_, 30 nm), and TiO_2 _(40 nm) on BRL 3A liver cells, at concentrations up to 250 μg/ml, following a 24 hour exposure. Both particle types were able to decrease cell viability, but only at the highest concentration tested.

Linnainmaa et al. [38] assessed the toxicity of TiO_2_, in NP (rutile and anatase (20 nm)), and microparticulate (170 nm) forms) to rat liver epithelial cells, in the presence and absence of UVA light. No cytotoxicity or chromosomal damage was observed within cells exposed to all TiO_2 _particle types in the presence or absence of UVA.

The two studies that investigated the toxicity of TiO_2 _particles to the liver, suggest that TiO_2 _exhibits a low level of toxicity to liver cells, but this would require further investigation to be confirmed, due to the limited number of liver cell targets considered within the available studies. The consequences of liver exposure, to TiO_2 _are of particular interest due to evidence of preferential NP accumulation within this organ (see earlier).

### Cardiovascular models

The interaction of TiO_2 _with endothelial cells that line blood vessels has been a focus of investigations, to determine the consequences of particle transport with the blood. In addition, the impact of TiO_2 _on cardiomyocyte function has been investigated.

Peters et al. [39] evaluated the impact of a NP panel (including TiO_2_, 70 nm) on HDMEC endothelial microvascular cell viability and function. Cells were exposed to TiO_2 _at concentrations ranging from 0.5 to 50 μg/ml, for up to 72 hours. NPs were internalised by cells into cytoplasmic vacuoles, but minimal toxicity was associated with this, therefore highlighting that particle uptake does not necessarily impact on cell function. Consequently, TiO_2 _was relatively non-toxic, with no cytotoxicity, and minimal IL-8 release stimulated by exposure.

Courtois et al. [40] determined the impact of TiO_2 _(15 nm or 0.4 μm diameter) exposure on nitric oxide (NO) mediated relaxation of pulmonary arteries *in vitro*. Pulmonary arteries were exposed to particles for 24 hours (at concentrations up to 200 μg/ml), in the presence or absence of vascular relaxants. It was found that urban particulate matter was able to impair NO-dependent relaxation within intrapulmonary arteries *in vitro *and *in vivo *(following intratracheal exposure). In contrast, manufactured NPs, including TiO_2 _did not exhibit this effect. Therefore this study suggested that the pulmonary circulation was not affected by TiO_2 _exposure, and that cells exhibited differed in their sensitivity to particles.

Helfenstein et al. [41] observed that TiO_2 _NPs (up to 125 μg/ml, 20-30 nm diameter) were able to affect cardiomyocyte electrophysiology, enhance ROS production, and reduce myofibril organisation. A panel of particles was tested, of which diesel exhaust particles (DEPs) were demonstrated to exhibit the greatest toxic potency, and SWCNTs were found to have limited toxicity in comparison. The findings from this study suggest that cardiac cells may be damaged by TiO_2 _or DEP exposure, and so were unable to function normally *in vitro*.

A limited number of studies have been conducted to determine the impact of TiO_2 _particles within *in vitro *models of the cardiovascular system. However, the findings suggest that TiO_2 _may promote an inflammatory response within blood vessels which has the propensity to stimulate or aggravate cardiovascular disease. Consequently, the ability of TiO_2 _to contribute to inflammatory mediated disease requires further consideration *in vivo*. In addition, the ability of TiO_2 _to negatively impact of cardiomyocyte function has the ability to disturb normal cardiac electrophysiology. Again, further studies, *in vivo*, would be required to verify this observation.

### CNS models

Long et al. [42] established the contribution of oxidative stress to the neurotoxicity of TiO_2 _NPs (30 nm diameter, but particles agglomerated in culture medium). BV2 microglia cells were exposed to TiO_2 _for periods of up to 120 mins, at concentrations ranging from 5 to 120 ppm, and ROS production determined. The oxidative response mediated by TiO_2 _within microglia had two components. Firstly, a rapid increase in ROS production was observed at 1-5 minutes, and termed an oxidative burst. This was followed by a greater, sustained ROS release from 60-120 minutes. It is also relevant, that despite having a primary particle size of 30 nm, the particles were observed to aggregate within the cell culture medium, which increased with increasing concentration, and this appeared to promote ROS production, perhaps due to the greater uptake of larger structures by phagocytosis. Internalised particles were found within the cytoplasm. Mitochondria located in the vicinity of the internalised aggregates were found to be swollen and disrupted, which was postulated by the authors to promote an apoptotic or necrotic response.

Similarly, Long et al. [43] investigated the neurotoxicity of nanoparticulate TiO_2 _(up to 120 ppm). It was illustrated that particle aggregates were phagocytosed by BV2 microglial cells, and contained within membrane bound vesicles. ROS production by cells was associated with particle exposure, and stimulated the upregulation of genes involved with inflammation, apoptosis, and cell cycling, and a down-regulation in energy metabolism. It was therefore evident that increased ROS production as a consequence of TiO_2 _exposure also triggered cytotoxicity via apoptosis. However, TiO_2 _exposure was non-toxic to N27 neuronal cells, following a 72 hour exposure, despite being internalised. These results reinforce the finding that particle internalisation does not necessarily equate to the initiation of a toxic response within cells, and that cell types differ in their susceptibility to particle toxicity. In contrast there was evidence of neurone loss within primary cultures of rat striatum within 6 hours of exposure, which was suggested to occur via microglia mediated ROS production.

Information regarding the neurotoxicity of TiO_2 _is severely lacking, and restricted to the work conducted by one research group. However, it is evident that the response is likely to be dictated by the cell type under investigation. Again, an oxidant driven response appears to be integral to particle toxicity. At present, there is insufficient evidence to make generalisations regarding TiO_2 _mediated neurotoxicity.

### Kidney models

Only one study was found that assessed the toxicity of TiO_2 _NPs to the kidneys, *in vitro*. L'azou et al. [44] investigated the effect of TiO_2 _(15 or 50 nm) on IP15 mesangial, and LLC-PK_1 _proximal tubular epithelial renal cells (at concentrations up to 512 μg/ml, for 24 hours). The different cell types exhibited different sensitivities to the toxicity of TiO_2_. No cytotoxicity was observed within IP15 cells on exposure to TiO_2_. However, TiO_2 _elicited a cytotoxic response within LLC-PK_1 _cells, suggesting that this cell type was more sensitive to TiO_2 _toxicity. This was postulated to derive from their high endocytic activity, and therefore greater internalisation of particles. The cytotoxic response was also observed to be size dependent, with a greater response exhibited by smaller particles. TiO_2 _(15 nm) was also able to induce morphological changes within both cell types (namely cell shrinkage, and detachment), and was internalised into cytoplasmic vacuoles. TiO_2 _was unable to induce an increase in ROS production. However, 13 nm carbon black NPs were consistently recognised as being more toxic, in terms of cytotoxicity, and ROS production, which was evident in both cell types. Toxicity to renal cells was therefore observed to be particle size, particle composition, and cell type specific.

The findings from the only study identified as studying the toxicity of TiO_2 _particles to the kidneys, suggest that the response of the individual cell populations vary. However, further investigations would be required to identify if the finding was applicable to other particle types, and if the same response is evident *in vivo*. Therefore it is difficult to draw definitive conclusions about particle toxicity to the kidney, due to insufficient data being available.

### Immune system models

It is acknowledged that macrophages are primarily responsible for the clearance of particles at sites of exposure, and accountable for the accumulation of particles within different target sites, which is anticipated to derive from their role within the reticuloendothelial system. The consequences of particle uptake by macrophages is therefore of interest, particularly the initiation of an oxidative driven or inflammatory response, and impact on their phagocytic function. In addition, the ability of particles to affect other immune cell populations, such as lymphocytes, is of interest as particles would be expected to encounter these cells within the blood.

Renwick et al. [45] determined the impact of particle exposure on the phagocytic activity of macrophages. It was observed that TiO_2 _(and CB) particles were able to impair J774.2 macrophage cell phagocytic activity (indicated by the uptake of 2 μm latex beads), in a concentration dependent manner. TiO_2 _NPs (29 nm) were more effective at inhibiting macrophage phagocytosis than their microparticulate (250 nm) counterparts, and the same response was exhibited by CB particles. It is likely, that the impairment of phagocytosis occurs as a consequence of the fact that cells are overwhelmed by the TiO_2 _particle burden, and are therefore unable to further internalise the latex particles. This was suggested to be the case, as an increase in particle-laden macrophages correlated with a decrease in phagocytic cells. The impairment in phagocytic function of macrophages would be anticipated to impact on the clearance of particles, thus increasing their retention, and thereby prolonging their interaction with cells, and enhancing their propensity for eliciting damage.

Afaq et al. [46] demonstrated that alveolar macrophages were increased in rats on intratracheal exposure (2 mg/rat, with observations made for a period of up to 16 days) to TiO_2 _NPs (<30 nm), which was maximal at 8 days post exposure. The exposure of alveolar macrophages to TiO_2 _was associated with lipid peroxidation, glutathione depletion and enhanced hydrogen peroxide production, illustrating the oxidative response that developed. Exposure was also associated with cytotoxicity.

Kang et al. [47] determined the mechanisms underlying TiO_2 _(25 nm) NP toxicity within lymphocytes, at concentrations ranging from 20 to 100 μg/ml, for up to 48 hours. A particular focus of the study was on the oxidative potential of TiO_2_. A dose and time dependent decrease in cell viability was observed. TiO_2 _NPs also exhibited a genotoxic effect, whereby the frequency of micronuclei formation increased, which insinuated chromosomal damage occurred on exposure of cells to TiO_2_. DNA damage was also observed, using the Comet assay. Importantly, the pre-treatment of cells with the antioxidant N-acetyl cysteine (NAC), decreased TiO_2 _mediated DNA damage, thereby inferring that the response was ROS mediated. ROS production was subsequently confirmed to be increased by TiO_2 _exposure. The DNA damage inflicted by TiO_2 _resulted in the activation of a protective response in the form of increased levels of p53 protein, highlighting an attempt of the cells the repair the damage mediated by TiO_2_. TiO_2 _mediated increases in ROS production, and therefore DNA damage, was thought to be central to its toxicity within lymphocytes.

Investigation of the impact of TiO_2 _particle exposure on immune cell function is lacking. Alveolar macrophages are recognised as being integral to the removal of particles from sites of deposition, and are, in the main, responsible for the accumulation of particles within different target sites. It is therefore of concern that particles appear to impair macrophage phagocytosis, and this should be considered further in future experiments. It is also suggested that particles initiate an oxidative driven response within immune cells that may have inflammatory and/or cytotoxic consequences.

## The biological mechanisms driving TiO_2 _nanoparticle toxicity

### TiO_2 _mediated inflammatory responses

Inflammatory responses have been illustrated as being a prominent feature of a number of studies that investigate the toxicity of nanoparticles such as TiO_2_. *In vivo*, the infiltration of neutrophils has been repeatedly demonstrated to characterise the inflammatory response to TiO_2_ NPs [3,6], in addition to elevated, particle laden macrophage numbers [10,12]. This is likely to be mediated by increases in inflammatory mediators, such as IL-8, and TNFα that have been observed *in vivo *[12] and *in vitro *[18]. The inflammogenic nature of TiO_2 _has been observed within a number of target sites including the lung, brain, and cardiovascular system, and so is not an organ specific observation. Inflammation has been demonstrated to be recurrently associated with TiO_2_exposure, with its manifestation evident within different models, in response to different TiO_2 _samples. Therefore, in general the inflammatory response that manifest is not organ, or particle sample specific.

### TiO_2 _mediated oxidative responses

A focus of a number of investigations has been to identify the contribution of oxidative stress to the inflammatory and cytotoxic responses elicited by TiO_2_. Studies previously discussed found that oxidative stress was a prominent feature of the response to TiO_2 _NPs. These include evidence of increased ROS production, depletion of cellular antioxidants, increase in oxidative products (such as lipid peroxidation) or evidence that toxicity is diminished on pre-treatment with antioxidants. These observations have been made in a variety of cell types, including for example; lung epithelial cells [26], fibroblasts [35], and microglia [42]. A summary of the oxidative responses mediated by TiO_2 _can be found in table 2.

**Table 2:** The oxidative potential of TiO_2_

Paper	Particle	Model	Endpoints Assessed	Observation	Conclusion
Afaq et al., [46]	TiO_2 _(<30 nm)	Response of primary alveolar macrophages (following intratracheal exposure of rats)	Glutathione peroxidase, glutathione reductase, glutathione-s-transferase activity Intracellular GSH Lipid peroxidation (thiobarbituric acid reactive substances measured) H_2_O_2 _production Cytotoxicity (LDH assay)	Decreased GSH Increased lipid peroxidation Increased H_2_O_2 _(indicative of respiratory burst) Increased glutathione peroxidise & glutathione reductase Decreased cell viability	An oxidant driven inflammatory, and cytotoxic response was observed within macrophages on exposure to TiO_2_

Dunford et al., [58]	TiO_2 _(extracted from commercially available sunscreens)	DNA oxidative damage (plasmid DNA & within MRC-5 fibroblasts)	Oxidation of organic material (phenol) Plasmid DNA (*in vitro*) Comet assay (MRC-5 cells) (all experiments conducted in sunlight illuminated conditions)	TiO_2 _stimulates oxidation of organic materials (due to production of hydroxyl radicals) & strand breaks in plasmid DNA. DNA damage decreased with free radical quenchers (mannitol & DMSO) - illustrates that it is oxidant driven DNA damage observed in comet assay & is oxidant driven	Oxidative damage to DNA by TiO_2_

Gurr et al., [28]	TiO_2 _(10, 20 or >200 nm)	BEAS-2B epithelial cells	Oxidative DNA damage (Comet assay) Lipid peroxidation (MDA) NO and H_2_O_2 _production Cell viability (MTT assay)	Increased DNA damage Increased lipid peroxidation Increased NO & H_2_O_2_ Decreased cell viability Responses only for 10 & 20 nm NPs	Oxidative stress induced appears to be size dependent, and has genotoxic and cytotoxic consequences

Jin et al., [35]	TiO_2 _(20-100 nm)	L929 fibroblasts	Cell viability (MTT DH assays) ROS production (dichlorofluorescein (DCFH) assay) GSH & SOD cell levels	Decreased cell viability Increased ROS production Decreased GSH and SOD	TiO_2 _mediated oxidative stress is related to a loss of cell viability

Kang et al., [49]	TiO_2 _(21 nm & 1 μm)	RAW 264.7 macrophages	Intracellular ROS generation (DCFH assay & dihydroethidium staining) Cell viability (LDH) Cytokine production MAPK signalling pathway activation	No loss in cell viability Increased ROS production (greater for NPs) Increased MIP-2 and TNFα ERK1/2 phosphorylation (part of MAPK pathway)	NPs stimulate the production of ROS that, in turn activate a signalling cascade (involving ERK1/2) to promote the development of an inflammatory response

Karlsson et al., [57]	CuO (42 nm), ZnO (71 nm), TiO_2 _(63 nm), Fe_3_O_4 _(20-30 nm)	A549 lung epithelial cells	Cell viability (trypan blue) ROS production (DCFH assay) Comet assay	Cytotoxicity greatest for CuO CuO increased ROS and elicit DNA (oxidative mediated) damage -Fe_3_O_4 _did not elicit toxicity	CuO most toxic NP, via an oxidative mechanism, but the release of ions may be responsible for the observed toxicity Metal oxide NPs vary in their ability to elicit oxidant mediated damage

Long et al., [43]	TiO_2_	BV2 microglia, N27 neurones	ROS production (DCFH) H_2_O_2 _production (Image-IT LIVE fluorescent probe) Superoxide production (MitoSOX fluorescent probe) Apoptosis (capase 3/7 activity & nuclear staining)	Increased ROS production Increased H_2_O_2 _(rapid response, 1-5 mins) Increased superoxide (later response, 30 mins onwards) Increased Apoptosis -Toxicity only evident in BV2 cells	Neurotoxicity mediated by TiO_2 _is oxidant mediated Cell dependent sensitivity to toxicity observed.

Lu et al., [51]	TiO_2_	BSA	Protein nitration (detected spectrophotomically & western blotting) (experiments conducted with UV irradiation)	Protein nitration is crystal form dependent Antioxidants prevent against protein nitration	Protein nitration is crystal form and light dependent

Park et al., [26]	TiO_2 _(21 nm)	BEAS-2B lung epithelial cells	Cell viability (MTT assay) ROS production (DCFH assay) GSH depletion Apoptosis (caspase-3 assay & chromosome condensation) Gene expression (RT-PCR)	Increased cytotoxicity Increased ROS production Decreased GSH Increased apoptosis Increased expression of oxidative stress (e.g. catalase, HO-1, glutathione-S- transferase) & inflammatory (IL-1, IL-6, IL-8, TNFα) genes	TiO_2 _NPs induce oxidative stress in cells, which is responsible for the observed inflammatory & cytotoxic (via apoptosis) responses

Sayes et al., [71]	TiO_2 _(in various crystal forms)	HDF (dermal fibroblasts) & AA549 (lung epithelial) cells	Cytotoxicity (LDH, MTT & live/dead assays) Inflammation (IL-8 production) Particle suspension ROS *ex vivo *production	Increased cytotoxicity Increased ROS (*ex vivo*) production Increased IL-8 production -Response dependent on crystal form	Toxicity exhibited by TiO_2 _is phase dependent, and involves, oxidative, inflammatory and cytotoxic components

Wang et al., [17]	TiO_2 _(in rutile (80 nm) & anatase (155 nm) forms)	Nasal Instillation (mice)	Enzyme activity (gluthathione peroxidise, catalase, SOD, glutathione-S-transferase) GSH levels Lipid peroxidation (MDA) Protein oxidation (protein carbonyl formation) (All responses evaluated in the brain)	Increased MDA Increased catalase Decreased SOD Increased protein oxidation -No changes in other markers	TiO_2 _distributes within the brain and elicits oxidative damage, which is dependent on the crystal phase of the particles

Xia et al., [50]	TiO_2 _(11 nm) (also ZnO (13 nm) & CeO_2 _(8 nm))	RAW 264.7 macrophages & BEAS-2B lung epithelial cells	Cytotoxicity (Propidium iodide & MTS assays) Intracellular ROS production (DCFH assay), and HO-1 antioxidant expression. Pro-inflammatory signalling cascade activation (nfKB) and intracellular calcium concentration. Cytokine production (TNFα & IL-8)	No increase in cytotoxicity, ROS generation or inflammation was observed	The most toxic particle in the panel was ZnO. Toxicity was absent for TiO_2_.

It is acknowledged that the development of a moderate level of oxidative stress is connected to the initiation of inflammatory responses through the activation of ROS sensitive signalling cascades [48]. Consequently Kang et al. [49] compared the ability of nano (21 nm) and microparticulate (1 μm) forms of TiO_2 _(0.5-200 μg/ml) to initiate an oxidative response within RAW 264.7 macrophages, following exposure for up to 24 hours. No alterations in cell viability were observed on exposure of cells to both forms of TiO_2_. This finding is of importance, as when assessing the processes that underlie particle toxicity, sub-lethal concentrations are required. ROS production was increased by TiO_2_, and was greatest in magnitude for the NPs. The ROS sensitive mitogen-activated protein kinase (MAPK) signalling pathway was activated by TiO_2 _exposure (indicated by ERK1/2 phosphorylation), which was suggested to initiate the increase in pro-inflammatory mediator production (TNFα, MIP-2). Within the endpoints measured, the response exhibited by NPs was consistently greater than that of their larger counterparts, when administered at an equivalent mass basis. The oxidative stress paradigm therefore held true within this study; specifically that particle mediated ROS production (at a moderate level) stimulates signalling cascades that promote the activation of transcription factors that, in turn, initiate an inflammatory response. Accordingly, the ability of particles to promote ROS production is thought to be central to their ability to regulate inflammatory responses.

In line with these findings, Xia et al. [50] investigated whether TiO_2 _(11 nm), ZnO (13 nm), and CeO_2 _(8 nm) oxidative stress development or particle dissolution contributed to their toxicity. RAW 264.7 macrophages and BEAS-2B epithelial cells were exposed to the NP panel for up to 15 hours, at concentrations up to 60 μg/ml. However, only ZnO particles were capable of inducing cytotoxicity, within both cell types. In addition, ZnO stimulated an increase in ROS production, which in turn, increased HO-1 expression, and activated the JNK signalling pathway, which paralleled the release of IL-8 and TNFα. Increased calcium release was also mediated by ZnO, and was associated with mitochondrial damage. These findings therefore imply that ZnO elicits an oxidant driven response that was responsible for the initiation of an inflammatory and cytotoxic response, which was absent for TiO_2 _and CeO_2_. This is an important finding, as contrary to evidence that TiO_2 _can exhibit an oxidative mediated response, this does not always transpire, and therefore the experimental model and TiO_2 _sample may be influential to the observed response. On the contrary, CeO_2 _was able to stimulate a cytoprotective response, whereby pre-treatment of cells with CeO_2 _protected against diesel exhaust particle mediated cell damage (which is known to be oxidant driven). All particle types were internalised by an endocytic mechanism, however, only ZnO accumulated within lysosomes, which was suggested to drive their ability to inflict oxidative injury, and promote particle dissolution. ZnO also induced ultrastructural alterations, including nuclear fragmentation, apoptotic body formation and mitochondrial disappearance. ZnO was therefore recognised as being the most toxic particle within the panel, however this was thought to be accounted for, in part, by their dissolution and release of Zn^2+ ^ions. The toxicity of the metal oxide particles was therefore suggested to be driven by their oxidant properties, which was dependent on their composition, dissolution and intracellular fate. The composition of NPs has been demonstrated to be integral to their toxicity within this study, and thus not all metal oxide particulates are expected to behave similarly.

Lu et al. [51] had a slightly different focus and determined the impact of TiO_2 _(0.2-3 mg/ml) on protein tyrosine nitration (using bovine serum albumin (BSA) as a model protein). Protein nitration was associated with TiO_2 _exposure, in the presence of UV light, with anatase TiO_2 _samples being more photoactive than rutile forms. Antioxidants, such as GSH and vitamin E were able to prevent against the protein nitration observed. To determine if proteins contained within mouse skin were targets for TiO_2 _mediated nitrosation, similar tests were conducted using mouse skin homogenate. Again, anatase forms of TiO_2 _were observed to exhibit a greater propensity to nitrosate proteins. This study therefore highlighted the photocatalytic activity of TiO_2_, and the superior toxicity of anatase forms, and demonstrated the ability of TiO_2 _to induce nitrative stress.

Oxidative responses exhibited by TiO_2 _particles have been a focus of a number of studies, and its recurrence has prompted the suggestion that oxidative stress drives the inflammatory and cytotoxic responses evident. It is relevant that the level of oxidative stress, which is inevitably related to the duration or concentration of particles administered, drives the nature of the response that follows particle exposure. Specifically, at moderate levels of oxidative stress, inflammatory responses may be stimulated due to the activation of ROS sensitive signalling pathways [48]. At higher levels of oxidative stress, cytotoxicity is evident, as cells are damaged by an overwhelming burden of ROS [48].

### Uptake of TiO_2 _into cells

The clearance of TiO_2 _particles by phagocytic cells has been a particular focus of studies, due to their ability to remove particles from the exposure sites, circulation or secondary target sites. However, a number of non-phagocytic cell populations also have the propensity to internalise particles. Considering the internalisation of particles by cells is of relevance as particle uptake may enhance their toxicity due to their interference with normal cellular physiology and function. However, it is also possible that particles act from outside the cell to elicit toxicity that is mediated by interactions of particles with the cell surface. Furthermore, particles can be internalised by cells and not impact on cell function, which is likely to be driven by the intracellular location in which they accumulate or the extent of uptake.

Stearns et al. [52] demonstrated that 50 nm TiO_2 _(40 μg/ml) was internalised by alveolar A549 epithelial cells into membrane bound vesicles after 3, 6 and 24 hour exposures. However, it was observed that the uptake of particles by these cells was limited to their aggregated form. TiO_2 _internalisation was suggested to be mediated via phagocytosis, due to the fact that plasma membrane projections surrounded and engulfed the particles prior to uptake.

A focus of a number studies has been to reveal the contribution of macrophage populations to the clearance of particles, particularly within the lung. However, Geiser et al. [53] argued that alveolar macrophages were not primarily responsible for the uptake and clearance of NPs within the lung *in vivo*. Rats were exposed to TiO_2_, via inhalation (0.1 mg/m^3^), and alveolar macrophages were isolated, and the internalisation of particles assessed, 1 or 24 hours post exposure. Large TiO_2 _particles (3-6 μm) were more effectively cleared by alveolar macrophages, than their smaller (20 nm) counterparts. Therefore TiO_2 _NPs were able to bypass the important alveolar macrophage mediated clearance mechanism within the lung, due to their small size. These findings could be expected due to the known size limitations of uptake processes such as phagocytosis, which is thought to be restricted to substances that are 1 to 5 μm in size [54]. The ability of TiO_2 _NPs to evade phagocytosis was confirmed by the findings that they were not enclosed by a vesicular membrane equivalent to that surrounding the larger TiO_2 _particles. Therefore it was suggested that phagocytosis was not responsible for NP uptake, and instead it was suggested that a sporadic, non-specific mechanism of uptake enabled NP uptake by macrophages. Alternatively it was suggested that NPs were internalised unintentionally when macrophages phagocytosed other material. The findings of Geiser et al. [53] are also able to provide an explanation for the finding that only TiO_2 _agglomerates, and not individual NPs, were internalised by A549 cells *in vitro *[52]. This was also recognised by Stearns et al. [52] who highlighted that there was limited evidence of the uptake of individual particles by cells. As Stearns et al. [52] utilised epithelial cells, and Geiser et al. [53] used macrophages which are professional phagocytes, it is relevant that the processes that drive particle uptake may be cell specific, and reliant on their function.

Rothen-Rutishauser et al. [55] utilised an *in vitro *airway wall model (triple cell co-culture), containing macrophages, epithelial, and dendritic cells to determine the translocation of particles between the different cell types, and to assess the intracellular fate of particles. Membrane-bound aggregates (>0.2 μm) of TiO_2 _were evident within all cell types. However, smaller aggregates (<0.2 μm) were apparent within the cell cytoplasm, but were not membrane bound, suggesting that the entry mechanism of particles was size dependent. However, it is relevant that the behaviour of gold, and polystyrene particles was also assessed, and it was found that their intracellular location differed, which implied that different NPs are internalised by different mechanisms or follow different intracellular trafficking processes.

The uptake of TiO_2 _by a variety of cell types has been demonstrated on numerous occasions. The consequences of particle internalisation are anticipated to be oxidative and cytotoxic in nature. It is anticipated that particle physicochemical properties may influence their internalisation by cells. Accordingly, the size and surface charge of particles has the ability to influence their uptake by cells. In addition, particle size and the cell type under investigation also have the potential to determine the mechanism of uptake and intracellular fate of particles.

### Genotoxicity of TiO_2_

Evidence of genotoxicity has been previously encountered within a number of studies; micronuclei development is associated with TiO_2 _exposure, which is indicative of chromosomal damage, DNA damage has also been observed in response to TiO_2 _particulate exposure.

Rahman et al. [56] investigated the potential for TiO_2 _to elicit DNA damage within SHE fibroblasts. Micronuclei were evident within cells exposed to nano (<20 nm), but not micro (>200 nm) TiO_2 _particles (exposed at concentrations up to 10 μg/cm^2^, for up to 72 hours), which insinuates that chromosomal damage has occurred. The NPs also triggered the induction of apoptosis within cells, which is recognised as a common response to DNA damage.

Karlsson et al. [57] evaluated the ability of a variety of metal oxide NPs (copper oxide (CuO), ZnO, TiO_2_, Fe_3_O_4_) to induce oxidative stress and DNA damage within A549 lung epithelial cells, at concentrations of up to 80 μg/ml for a period of up to 18 hours. CuO and ZnO were able to decrease cell viability, but TiO_2 _and Fe_3_O_4 _were not capable of eliciting such an effect. In addition, all metal oxide particles, except Fe_3_O_4 _were able to elicit DNA damage, as determined by the Comet assay. The CuO NPs were consistently demonstrated to be the most toxic particle within the panel, which was thought to be drive by their ability to induce oxidative stress, and in turn DNA damage, which prompted cell death. It was suggested that the release of Cu^2+ ^ions may contribute to the response, but are not solely accountable for the toxicity of CuO NPs. Importantly, this study illustrated that not all metal oxides behave similarly, and that their dissolution was able to contribute to their toxicity.

Dunford et al. [58] investigated the DNA damage inflicted by a panel of TiO_2 _containing, commercially available sunscreens on fibroblasts, when exposed for a period of up to 60 minutes. It was demonstrated that, on sunlight illumination, the ability of TiO_2 _to cause DNA damage was enhanced in plasmid DNA and cells. Anatase forms of TiO_2 _were more effective at inducing damage than rutile forms. Mannitol (an antioxidant) was able to protect against DNA damage, therefore implying that TiO_2 _mediated ROS production is central to its genotoxic potential. Consequently, absorption of UV light by TiO_2 _is thought to stimulate ROS generation, which, in turn, leads to DNA damage.

Nakagawa et al. [59] investigated the genotoxicity of a panel of TiO_2 _NPs (in rutile and anatase forms, ranging from 0.021 to 0.255 μm in diameter). A number of different *in vitro *genotoxicity tests were utilised; a microbial mutation assay (*Salmonella typhimurium*), the Comet assay to detect DNA damage (mouse lymphoma L5178Y cells) and a chromosome aberration assay (Chinese hamster CHL/IU cells). TiO_2 _was used at concentrations up to 3200 μg/ml, with an exposure duration of up to 24 hours, and experiments were conducted in the absence or presence of UV light. Without UV light, TiO_2 _NPs induced no, or very limited genotoxicity. However in the presence of UV light TiO_2 _elicited DNA damage and chromosome aberrations (but no gene mutations) that was greatest for anatase forms. The phototoxicity of TiO_2 _was therefore realised in this study.

Theogaraj et al. [60] investigated the UV dependence of the genotoxic potential of a panel of TiO_2 _NPs (in rutile and anatase forms, all of which had a diameter of <60 nm). CHO cells, were exposed to TiO_2 _at concentrations up to 5000 μg/ml for a period of 3 hours. In contrast to previously discussed investigations, no chromosomal alterations were observed within exposed cells, in the presence or absence of UV light.

In a different approach, Driscoll et al. [61] determined if there was a relationship between the inflammatory and genotoxic potential of several particles; namely ufCB (14 nm), TiO_2 _(0.18 μm) and α quartz (0.9 μm). Rats were exposed to particles via intratracheal instillation, at a dose of 10 or 100 mg/kg, and analysis conducted 15 months post exposure. All particle types induced the infiltration of neutrophils into the lungs, indicating that particles could induce an inflammatory response. Hypoxanthine-guanine phosphoribosyl transferase *(hrpt) *gene mutation frequency was increased within alveolar type 2 cells, on exposure to particles. This response was replicated *in vitro*, following the exposure of RLE-6TN alveolar epithelial cells to BAL cells isolated from particle treated animals. It was thus suggested that BAL cell derived ROS were responsible for the mutagenic effects that transpired, and they were suggested to derive from neutrophil activity. Therefore the particle mediated neutrophillic driven inflammatory response within the rat lung, was postulated to increase the frequency of mutations within alveolar epithelial cells, which was a dose and material dependent finding. Accordingly, quartz was consistently found to elicit the greatest genotoxic response, followed by carbon black and then titanium dioxide, and this was related to their inflammogenic potential. Therefore, the particles themselves are not thought to be inherently genotoxic, but the inflammatory response, and in particular neutrophil presence (and therefore increased oxidant burden) instigated is anticipated to mediate this effect.

Concern regarding the genotoxic potential of TiO_2_, has also emanated from findings that have illustrated that tumours develop *in vivo *following a chronic exposure regime ([13] see earlier). However, as stated previously this is very much dependent on the exposure conditions, and in particular the exceptionally high doses that were administered within this model.

The ability of TiO_2 _NPs to inflict DNA damage has been observed on numerous occasions, and is thought to be driven by particle mediated ROS production, with cell death often stimulated as a protective response. In addition, the manifestation of genotoxic events, as a secondary consequence of an inflammatory response is also evident, and requires further investigation. The appearance of TiO_2 _mediated genotoxicity appears to be influenced by the crystal form of the sample, particle dissolution and light conditions. The finding that chronic exposure to TiO_2 _can induce tumours *in vivo *(under extreme exposure conditions), also implies that the genotoxicity exhibited by TiO_2 _is not limited to *in vitro *investigations.

### Reproductive toxicology of TiO_2_

Evaluation of TiO_2 _NP effects on the reproductive system is limited to one *in vitro *study.

Komatsu et al. [62] determined the potential for TiO_2 _NPs, DEP and ufCB to impair the function of male mouse reproductive system. This study evaluated the direct effect of NPs on testis-constituent cells, and examined the effect of TiO_2 _on mouse Leydig TM3 cells, the testosterone-producing cells of the testis. TiO_2 _NPs (25-70 nm) at concentrations 1 to 1000 μg/ml were examined and uptake into Leydig cells was detected using transmission electron microscopy (TEM) or field emission type scanning electron microscopy/energy-dispersive X-ray spectroscopy (FE-SEM/EDS). TiO_2 _was more cytotoxic to Leydig cells than the other carbon based particles used in the study. The proliferation of Leydig cells was suppressed transiently by treatment with TiO_2_. TiO_2 _NPs were taken up by Leydig cells, and in turn affected cell viability, proliferation and gene expression.

Only one available study examined the effects of TiO_2 _on male reproductive physiology. The literature highlights toxicity of TiO_2 _NPs to male Leydig; however investigations are limited in number and sample size, and therefore further studies would be required to confirm such a finding. No literature examining TiO_2 _NP effects on organs or cell types in the female reproductive system were found.

## Linking the physicochemical attributes of TiO_2 _particulates to their pathogenicity or toxicity

As TiO_2 _particulates exhibit great diversity, with regards to their size (and therefore surface area), composition, and crystal form, it is necessary to outline what properties are most influential in driving the toxicity of TiO_2_. However it is also relevant to highlight that the experimental set up is also able to influence the findings, including the choice of species or cell type, method of exposure, and particle dispersal (see later)

### Size dependency

Particle dimensions are recognised as being fundamental to their toxicity. This derives from the fact that NPs have been consistently demonstrated to be capable of eliciting more pronounced toxicity than their larger (microparticulate) counterparts. The size dependency of TiO_2 _toxicity has been frequently demonstrated [3,12,24,28,45,47], and appears to be applicable to a variety of TiO_2 _forms, and occurs regardless of the model used (table 3). However it is relevant that a high degree of particle aggregation and agglomeration is associated with TiO_2 _administration, and so exposure to particles is unlikely to occur in a 'nano' form. However, what seems to be critical to the toxic potential of particle samples is the size of particles that make up the agglomerates. It is relevant that the terms agglomeration and aggregation are often used interchangeably within the field of nanotoxicology. However, some authors have suggested that NP aggregation and agglomeration are distinct phenomena with agglomerates formed by clusters of particles that are held together by electrostatic interactions, whereas aggregates are formed from covalently fused or sintered particles that are not easily separated [63].

**Table 3:** A summary of the size dependent toxicity of TiO_2 _particulates

Paper	TiO_2 _Particle Size	Model	Findings
Ferin et al., [3]	21 nm 250 nm	Rats Intratracheal instillation Inhalation	Pulmonary inflammation greatest for NPs

Gurr et al., [28]	10-20 nm 200 nm	Bronchial epithelial cell line (BEAS-2B)	NPs exhibit oxidative damage that is absent with fine particles

Kang et al., [47]	21 nm 1 μm	RAW 264.6 macrophages	ROS production, ERK activation and pro-inflammatory mediator production (TNFα & MIP-2) greater for NPs

Renwick et al., [45]	29 nm 250 nm	J774.2 macrophages	NPs impair macrophage phagocytosis, which is not apparent for fine particles

Renwick *et al*., [6]	29 nm 250 nm	Rats intratracheal	NPs stimulate pulmonary inflammation (neutrophil infiltration), epithelial damage and cytotoxicity to a greater extent than their fine counterparts The phagocytic ability of macrophages was impaired with NP exposure but not fine particles

Wang *et al*., [24]	25, 80 nm 155 nm	Mice Oral administration	Toxicity (mainly observed within the liver & kidneys) was greater for NPs

Within the table NPs (equivalent to ultrafine particles, in terms of size) are defined as having a diameter that is less than 100 nm, and fine particles have a diameter of greater than 100 nm.

As discussed, the superior toxicity of nanoparticulate forms of TiO_2 _has been repeatedly documented. However, a study conducted by Dick et al. [64] illustrated that compared to other NPs such as nickel, cobalt or carbon black, TiO_2 _nanoparticles were less potent at inducing an inflammatory response within the lung, despite their similarity in surface area. This was assumed to be related to their lower capacity to generate free radicals, and therefore perhaps particle composition and surface chemistry are also important contributors to toxic responses [64].

Bermudez et al. [7] exposed rats to TiO_2 _via inhalation, to investigate their pulmonary toxicity. Despite the fact that the NPs had a primary particle size of 21 nm, the aerosol generated contained particle aggregates (1.37 μm). This is a common experience in the exposure of animals or cell culture models to nanomaterials, and is likely to be encountered within the exposure of humans. However, despite the formation of aggregates, the NPs still exerted toxicity. Similarly, aggregates of TiO_2 _NPs (1.44 μm) have been demonstrated to be more toxic than similarly sized aggregates of their larger counterparts [3]. However, Grassian, et al. [10] demonstrated that the properties of agglomerates of particles that formed during aerosol generation were dependent on the primary particle size. Specifically, 21 nm particle based agglomerates were less dense than their 5 nm counterparts whose agglomerates contained particles that were more tightly packed. It was found that 21 nm TiO_2 _was more toxic than 5 nm particles following inhalation and instillation exposure. The agglomeration state of particles was therefore suggested to dictate their toxic potential. Consequently, although 21 nm particles were larger, it is anticipated that they would form agglomerates that would more easily deagglomerate due to the weaker interactions that held the particles together. As a result, 21 nm particles would be available as smaller structures to stimulate an enhanced toxic response. Furthermore the aggregates formed were larger within inhalation preparations than intratracheal suspensions, and so this may explain why intratracheal exposures produced a greater toxic response.

The greater toxicity of NPs, compared to their larger counterparts, is anticipated to be driven by the greater surface area of smaller particles, when administered at an equivalent mass dose. In fact, the surface area of particles has been previously suggested to dictate the toxic potential of particles [65-67]. In line with this hypothesis, Sager et al. [68] addressed which dose metric was most influential in driving the toxicity of TiO_2_; the surface area or mass of particles administered. NPs (suspended in BALF) were observed to agglomerate to a diameter of 200-300 nm (despite having a primary particle size of 21 nm). This finding emphasises that particle exposure often does not occur in an individual particle form. Microparticle (1 μm) and NP particles were able to induce an inflammatory response (neutrophil infiltration, LDH activity, TNFα, MIP and IL-1β release), subsequent to intratracheal administration of rats. It was of interest that a higher mass of microparticulate TiO_2 _was required to obtain the same inflammogenic response as nanoparticle TiO_2_. Therefore, the inflammatory and cytotoxic potential of NPs was greater than that of microparticles, when administered at an equivalent mass dose. However, when the dose of particles administered was equivalent, in terms of surface area delivered (which necessitates the administration of a much higher mass of microparticles), both particle types behaved similarly. These findings suggests that the size, and surface area of particles is integral to their toxicity. Similarly, Sager et al. [69] determined whether the surface area of ufCB and TiO_2 _NPs drives their inflammatory potential. Particles were administered to rats via intratracheal instillation, and toxicological observations made up to 42 days post exposure. At equivalent surface area doses, both particle types induced an inflammatory response that was characterised by the infiltration of neutrophils at day 1 which was of a similar magnitude. However the response decreased with time for ufCB, but for TiO_2 _the response was sustained in nature. This pattern of response was similar for BALF protein levels. Consequently, the composition of particles may also contribute to their toxicity. This observation remains debatable as others have disputed this finding [11].

The size (and therefore surface area) of TiO_2 _nanoparticles is known to be fundamental to their toxicity. The aggregation and agglomeration state of nanoparticles is also likely to be influential to their toxicity. Consequently, it is often the case that although the primary particle size is stipulated by investigators; cells and animals are not exposed to the particles in this form, [27,32,33,35,42,43,52,70-72]. However it is often observed that agglomerates/aggregates of nanoparticles are more toxic than similarly sized agglomerates/aggregates of their larger counterparts. Therefore, the propensity of particles to aggregate has prompted investigators to prevent against its occurrence through the use of sonication, or inclusion of dispersants such as within particle suspensions, but this also needs to take into account what is relevant to human exposure.

### Influence of TiO_2 _particulate surface chemistry/modification/coatings to their toxicity

As for other nanoparticles, the surface of TiO_2 _particulates can be altered through the attachment of surface moieties, a process which is termed particle functionalisation. The surface of TiO_2 _particles can also be modified through coating with aluminium oxide, or silica, which has generally been encountered within sunscreens to enhance protection from UV radiation [9].

Warheit et al. [9] investigated the impact of surface treatments (alumina or silica) on TiO_2 _toxicity, within the lungs (see earlier). It was demonstrated that those exhibiting the greatest toxicity, were those that contained the highest aluminium oxide and/or silica content on the particle surface. However, overall it was observed that TiO_2 _particles induced low pulmonary toxicity, despite the ability of surface coatings to influence this.

Oberdorster [73] demonstrated that the surface properties of TiO_2 _were able to influence their toxicity. This was demonstrated via the intratracheal exposure of rats (500 μg/animal, for 24 hours) to TiO_2 _(20 nm) that remained uncoated (hydrophilic) or received a silane coating (hydrophobic). The samples varied, with regards to their inflammatory potency within the lungs, so that the uncoated particles induced a lower response, than their coated counterparts.

Hohr et al. [74] investigated the impact of surface properties on TiO_2 _toxicity. Microparticulate (180 nm diameter), uncoated and coated (via methylation) NPs (20-30 nm diameter) of TiO_2 _were exposed to rats, via intratracheal instillation (1 or 6 mg/rat), and analysis conducted 16 hours post exposure. Administration of all particle types stimulated the infiltration of neutrophils, with the effect most pronounced with administration of NPs. It is also of interest that the coated (hydrophobic) NPs tended to stimulate neutrophil recruitment to a lesser extent than the uncoated (hydrophillic) particles. Protein, LDH, TNFα and MIP-2 levels in BAL also exhibited a similar pattern, with regards to the potency of the different samples, but overall the impact of surface methylation on TiO_2 _toxicity was negligible. However, it was concluded that the toxicity of TiO_2 _was driven by the surface area of particles, and not their surface coating. Therefore, a stronger inflammatory response was associated with NPs compared with their larger counterparts and so the effect of methylation was negligible.

Singh et al. [75] determined the impact of TiO_2 _size (microparticles: 40-300 nm, NPs: 20-80 nm), surface modification (using methylation, to make particles more hydrophobic) and radical generating potential on their uptake and toxicity within A549 lung epithelial cells. TiO_2 _particles, regardless of their size or methylation were phagocytosed as clusters of particles. Internalisation of NPs via clathrin mediated endocytosis, was only apparent for small particle clusters that were less than 30 nm, illustrating that this may be a size dependent phenomenon. ROS generation, and IL-8 production exhibited by cells exposed to NPs was greater than that of microparticles, and the findings were unaffected by the methylation of the particle surface. An important observation was that both particle types were present in an aggregated form, and that the NPs were in a smaller size range (<500 nm) than their larger counterparts (2000-5000 nm). Therefore the superior toxicity of NPs held true, despite their tendency to aggregate. Therefore, even when in an aggregated form the toxicity of TiO_2 _particles was assumed to be primarily driven by their surface area.

Thevenot et al. [76] determined the impact of TiO_2 _NPs, with various surface modifications (-COOH, -OH or -NH_2 _functional groups), on the survival of a variety of cancer cell lines. Exposure concentrations were high (up to 10 mg/ml) and occurred for a duration of 3 to 24 hours. The cancer cell lines used were B16F10 and BF16F1 melanoma, Lewis lung carcinoma, JHU prostate cancer, and 3T3 fibroblast cell lines. The different cell lines showed different sensitivities to the cytotoxicity of TiO_2 _NPs. 3T3, B16F10 and BF16F1 cells were unresponsive to all types of TiO_2_. However, TiO_2 _decreased the viability of JHU and LLC cells in a dose dependent manner. In addition, by altering the surface chemistry of TiO_2 _particles, the toxicity of TiO_2 _could be modified. In general, NH_2_, and OH surface modified TiO_2 _exhibited greater toxicity than COOH modified TiO_2_. This study therefore illustrated that TiO_2 _toxicity was very much dependent on the cell type in question, the surface chemistry of TiO_2 _NPs, and concentration of particles used.

Rehn et al. [70] illustrated that unmodified, or silane coated forms of TiO_2 _were non toxic to the lungs. Rats were exposed to the different forms of TiO_2 _(20 nm) via intratracheal instillation (up to 1.2 mg/rat) and the inflammatory and genotoxic effects within the lung were determined, at 3, 21 and 90 days post exposure. All forms of TiO_2 _were able to stimulate a modest increase in neutrophils and macrophages within the lungs (at day 3), but this was not persistent in nature, and was not associated with TNFα production. In addition, no genotoxicity was evident with TiO_2 _exposure. In contrast, quartz induced a persistent and progressive inflammatory response, with a genotoxic element to the response also observed. However, the only genotoxic endpoint that was assessed was the detection of 8 oxy-guanine, so perhaps a more comprehensive genotoxic study should be considered for TiO_2_.

The modification of the surface of TiO_2 _particles is able to influence its toxicity, however, this is likely to be dependent on the modification, and cell type in question.

### Crystallinity

TiO_2 _exists in two main crystal phases, termed rutile and anatase. These TiO_2 _forms vary with regards to their crystalline structure and surface properties, which is responsible for differences within their toxicity [77]. Anatase has been demonstrated to be the most toxic form of TiO_2_, with a number of previously mentioned studies supporting this conclusion (see for example [17]). The photoactivity of TiO_2 _is also dictated by the crystal phase, and therefore surface characteristics of particles (such as oxygen vacancies on the particle surface), with anatase forms having a greater capacity to generate ROS on exposure to light (see later). A summary of the contribution of particle of crystallinity to TiO_2 _toxicity is demonstrated in table 4.

**Table 4:** The importance of crystallinity to TiO_2 _toxicity

Paper	TiO_2 _crystal form	Model	Finding	Toxic potency
Dunford et al., [58]	TiO_2 _extracted from sunscreens (content ranging from 50/50 anatase/rutile, to 100% anatase or rutile) Pure Anatase (100%) Pure Rutile (100%)	Oxidation of organic material (phenol) DNA plasmids *in vitro* Comet Assay (MRC-5 fibroblasts) (ALL conducted in presence of sunlight)	TiO_2 _stimulates oxidation of organic materials (due to production of hydroxyl radicals), on illumination Strand breaks in plasmid DNA. The damage suppressed by free radical quenchers (mannitol &DMSO) - illustrates that it is oxidant (hydroxyl) driven DNA damage observed in comet assay, and again is oxidant driven	Anatase > rutile. Derives from greater photocatalytic activity.

Lu et al., [51]	Pure anatase (5 nm) Pure rutile (50 nm) Anatase/rutile mixture (21 nm)	Protein tyrosine nitration (Conducted in presence of UV light)	TiO_2 _increased protein tyrosine nitration (indicative of oxidative and nitrative stress)	Anatase >anatase/rutile > rutile BUT other physicochemical differences such as size were, not controlled for, which may contribute to response

Nakagawa et al, [59]	Anatase form (21 nm) Anatase form (255 nm) Rutile form (255 nm) Rutile form (420 nm)	*In vitro *genotoxicity assays: Microbial mutation assay-*S. Typhimurium* Mammalian cell mutation assay (L5178Y cells) Chromosomal aberration assay (CHL/IU cells) (experiments conducted in presence or absence of UV light)	Weak genotoxicity in absence of UV light With irradiation, TiO_2 _particles were genotoxic in all tests	Anatase > rutile Phototoxic component to response 21 nm anatase sample most toxic (illustrates that size may also contribute to response)

Pan et al. [72]	Rutile (15 nm) Anatase (200 nm)	Human dermal fibroblasts Cell area, morphology & actin Cell proliferation Wound healing function Cell migration Particle internalisation	Cell morphology detrimentally affected and cell function impaired by TiO_2_	Anatase > rutile

Sayes et al., [71]	Pure anatase Anatase/rutile Pure rutile	Human dermal fibroblasts and lung carcinoma cells Cell viability (LDH, MTT) Inflammation (IL-8) *Ex vivo *ROS production	Cytotoxicity, ROS production & cytokine release is crystal phase dependent	Anatase greater than anatase/rutile > rutile Oxidant driven response & phototoxic component Size of particles did not contribute to the response

Wang et al., [17]	Rutile (80 nm) Anatase (155 nm)	Mice (intranasal) Particle distribution in brain Neurone morphology & toxicity Oxidative stress (lipid and protein oxidation) Neurochemical levels	Accumulation of particles in brain Both particle types translocate to brain Anatase elicit greater neurotoxicity	anatase > rutile

Warheit et al., [11]	Rutile Anatase/rutile	Rats (intratracheal) Inflammation (BALF cells, LDH, protein) Histopathology	Pulmonary inflammation (nature, and length of response dependent on particle sample)	Rutile/anatase > rutile Other factors such as particle size & agglomeration may also contribute to the response

Sayes et al. [71] assessed the *in vitro *toxicity of anatase (10 nm), rutile (5 nm) or anatase/rutile (3 nm) TiO_2 _samples to A549 epithelial cells and HDF fibroblasts, at concentrations ranging from 3 μg/ml to 30 mg/ml, for up to 48 hours. The level of cytotoxicity exhibited by the different particle types varied. Accordingly, anatase particles were the most cytotoxic, while the rutile TiO_2 _particles were the least toxic. This response was paralleled within the release of IL-8 from cells. TiO_2 _particle stimulated ROS generation and photoactivity was also greatest for pure anatase samples. Overall, the results implied that the anatase samples had the greatest toxic potency. Consequently, the authors suggested that it was the phase of TiO_2 _that was integral to its toxic potential. Therefore, despite the relatively large surface area of rutile TiO_2 _NPs (112 m^2^/g), their surface chemistry is thought to be less reactive than that of anatase samples (that had a larger surface area of 153 m^2^/g), and so rutile particles are less toxic. The findings also suggested that the ability of TiO_2 _particles to generate ROS, governs their cytotoxic and inflammatory potential, which is dictated by the crystal structure of TiO_2_, and hence toxicity is not solely driven by surface area. However, rutile NPs were observed to agglomerate to the greatest extent, and so this may also explain why this particle type was observed to be less toxic. Although the findings demonstrate that particle crystallinity is important to TiO_2 _toxicity, the relevance of the findings is questionable, as in general, toxicity was only observed at higher concentrations (greater than 300 μg/ml), which are not deemed to be physiologically relevant (see later).

Pan et al. [72] investigated the toxicity of a panel of TiO_2 _particles to primary human dermal fibroblasts. Uncoated rutile TiO_2 _(15 nm), polymer coated rutile TiO_2 _or uncoated anatase TiO_2 _(200 nm) particles were exposed to cells for up to 11 days, at concentrations up to 0.8 mg/ml. Uncoated rutile TiO_2 _caused alterations in cell morphology, so that cells had a smaller cell area, became elongated, and detached from the culture surface. Anatase TiO_2 _caused a greater magnitude of damage to cells, with severe morphological changes observed including breakage of actin filaments, and plasma membrane rupture within exposed cells. Rutile TiO_2 _did not impact on cell viability, but did slow cell proliferation rate but were internalised by cells, and were contained within cytoplasmic vesicles. Anatase TiO_2 _was internalised by cells, and could access the nucleus. H_2_O_2 _production by cells was greatest with anatase particles, implying that their ability to generate ROS was greatest within the TiO_2 _particle panel. Anatase particles were therefore more potent that rutile particles at inducing cell damage. Rutile particles were then coated in order to prevent against particle adhesion to the plasma membrane, and thus limit their potential for internalisation. Coated particles were not evident on the cell surface, or within the cell interior. Consequently, a polymer coating abolished particle toxicity, which was suggested to derive from the lack of particle adherence to the cell surface, and thus limited internalisation, so that they were unable to impact on normal cell function. This study therefore demonstrates that particle coatings, and therefore surface chemistry, are able to modify particle toxicity. However, this requires further investigation, and may be dependent on the surface coating and cell type in question.

The phase composition of TiO_2 _has been proposed to be influential in dictating the toxic potency of particles. This is likely to derive from their substantially different surface chemistry, with anatase forms exhibiting the greatest photocatalytic and biological activity. The importance of crystallinity, to the toxicity of particles has also been demonstrated for SiO_2 _(see for example Kaewamatawong et al. [78]).

Furthermore the ability of UVA or visible light to increase the toxic potency of TiO_2 _particles, through increased ROS production, has been a focus of a number of previously discussed studies [see for example [36,51,58]], but does not always transpire [38,60]. This phenomenon is anticipated to be exploited, particularly within the treatment of cancer [36]. The crystal form of TiO_2 _has been suggested to be responsible for driving the photoactivity of particles, due to the importance of surface properties [77].

## Quality of experiments conducted

The described studies vary greatly with regards to their experimental set up. It has been highlighted that the size of particles, their crystallinity, route of exposure, particle concentration, experiment duration and species used can all impact on TiO_2 _particle toxicity. Therefore, deciphering what particle attributes are most influential to TiO_2 _toxicity is challenging. For example, determining if the crystal phase of TiO_2 _is primarily responsible for driving particle toxicity is confounded by the fact that the different TiO_2 _samples under investigation will not just vary with regards to their crystal phase but also, for example, their size and surface area. Consequently, it is difficult to isolate specific particle properties that are responsible for TiO_2 _toxicity, and it is likely that a variety of factors act in concert to dictate the toxic response observed. In line with this, it is also vital for investigators to carry out relevant physicochemical characterisation of their particle sample. It is suggested that prioritising the assessment of particle size, surface area, composition (including surface chemistry), crystal structure, and aggregation tendency would be of greatest relevance, due to their apparent importance to particle toxicity. If this is routinely carried out, this will facilitate making comparisons between different investigations to dissect out what particle attributes are driving the toxicity of TiO_2_.

A frequent observation within existing studies, is that excessively high concentrations of particles have been administered to animals or cells. It is therefore essential that investigators consider the relevancy of particle concentrations that are utilised, and justify their use if they are excessively high, as otherwise the relevancy of experiments is questioned. This is important as when administered at a large concentration, overload effects dominate, and the inherent toxicity of particles cannot be distinguished. For example, *in vivo*, this derives from the fact that an excessive particle burden cannot be efficiency cleared by defence mechanisms, and so the toxicity of particles is enhanced. In addition, the use of high particle concentrations is particularly concerning when assessing the toxicity of TiO_2 _to secondary target sites, such as the liver, as these targets are anticipated to be exposed to smaller concentrations of particles, due to the limited translocation of particles into the circulation from exposure sites. However, in saying this, wide concentration ranges can provide valuable information regarding the dose dependency of particle toxicity, and can also allow for the generation of threshold doses. However, for ethical reasons, the use of high concentrations should be restricted for *in vivo *work.

Gaps in current knowledge have been identified within the review, and thus there are a number of areas that should be considered with priority in future investigations. There is a paucity of data relating to the systemic transfer of TiO_2 _particles following exposure via the lungs, skin and gut, and this should be a focus of future experiments. Studies have focused on the dermal and pulmonary toxicity of particles, but there is an absence of data on the consequences of exposure to the GIT, and within damaged/diseased skin. Other relevant target organs include the liver, kidney, cardiovascular system and brain which is required due to the fact that NPs are likely to become systemically or neuronally available. The liver could be highlighted as a priority due to the propensity of particles to accumulate in this organ.

## Conclusions

Due to historical reasons, a focus on the size (and surface area) dependence of TiO_2 _particulate toxicity has been repeatedly investigated, and confirmation that particle toxicity increases as particle size decreases has been consistent within wide ranging investigations. However, it has become evident that other physicochemical factors are able to contribute to TiO_2_ toxicity; including particle aggregation, crystal phase, and surface modification. The exposure method, dose administered, species used, cell type under investigation and light conditions also have the potential to impact on the toxicity of TiO_2 _particles, indicating that the experimental set up is also very influential to the toxicological observations. Therefore, making generalisations about TiO_2 _particulate behaviour should be approached with caution, and the findings from a limited number of studies should not be considered to be representative for TiO_2 _particles as a whole. This is important as particle characteristics and experimental set up influence the toxicological observations made.

The toxicity of TiO_2 _has been demonstrated to have inflammogenic, oxidative, and genotoxic consequences; with these endpoints considered to be inherently linked. It is of interest that the biological mechanisms identified as being responsible for driving the toxicity of TiO_2 _particulates is replicated within *in vivo *and *in vitro *settings. Cytotoxicity is also a common end point that is evaluated within studies, although the relevance of this is questionable (in terms of human exposure levels), except when establishing sub-lethal concentrations for subsequent studies that allow the identification of mechanistic processes that are responsible for toxicity. The ability of particles to exert toxicity at a variety of target sites is reliant on their transfer into blood, and this should therefore be a focus of future experimentation, as at this time, the systemic availability of TiO_2 _particles following exposure is uncertain. Accordingly, investigations into the toxicity of TiO_2 _via specific routes of delivery, or at particular cell and organ targets, are often insufficient in number to make definite conclusions about particle behaviour. In addition, the quality (including the concentrations used, experimental model), and relevancy of conducted experiments is an important consideration, which is of vital importance when considering the risk associated with TiO_2 _exposure.

## Competing interests

The authors declare that they have no competing interests.

## Authors' contributions

HJ and VS contributed to all sections. GH contributed to the reproductive toxicity section. FC aided in the editing of the paper. SP contributed to the literature search. SH was involved in the scientific management of the project. All authors read and approved the final manuscript.
